# A yeast fermentate improves gastrointestinal discomfort and constipation by modulation of the gut microbiome: results from a randomized double-blind placebo-controlled pilot trial

**DOI:** 10.1186/s12906-017-1948-0

**Published:** 2017-09-04

**Authors:** Iris Pinheiro, Larry Robinson, An Verhelst, Massimo Marzorati, Björn Winkens, Pieter Van den Abbeele, Sam Possemiers

**Affiliations:** 1 ProDigest, Technologiepark 3, 9052 Ghent, Belgium; 2 Embria Health Sciences, 2105 SE Creekview Dr, Ankeny, IA 50021 USA; 3 Center of Microbial Ecology and Technology (CMET), University of Ghent, Coupure Links 653, 9000 Ghent, Belgium; 4 Department of Methodology and Statistics, Faculty of Health Medicine and Life Sciences, University of Maastricht, Maastricht, 6200 MD The Netherlands

**Keywords:** Constipation, Gastrointestinal discomfort, GI transit time, *Saccharomyces cerevisiae*, EpiCor fermentate, Human study, Gut microbiome, Bacteroidetes, *Bacteroides*, *Prevotella*

## Abstract

**Background:**

Constipation and symptoms of gastrointestinal discomfort such as bloating are common among otherwise healthy individuals, but with significant impact on quality of life. Despite the recognized contribution of the gut microbiome to this pathology, little is known about which group(s) of microorganism(s) are playing a role. A previous study performed in vitro suggests that EpiCor® fermentate has prebiotic-like properties, being able to favorably modulate the composition of the gut microbiome. Therefore, the aim of this study was to investigate the effects of EpiCor fermentate in a population with symptoms of gastrointestinal discomfort and reduced bowel movements and to evaluate its effect at the level of the gut microbiome.

**Methods:**

This pilot study was performed according to a randomized, double-blind, placebo-controlled parallel design. Eighty subjects with symptoms of gastrointestinal discomfort and constipation were allocated to one of two trial arms (placebo or EpiCor fermentate). Randomization was done in a stratified manner according to symptom severity, resulting in two subgroups of patients: severe and moderate. Daily records of gastrointestinal symptoms were assessed on a 5-point scale, and also stool frequency and consistency were documented during a 2-week run-in and a 6-week intervention phases. Averages over two-week intervals were calculated. Constipation-associated quality of life and general perceived stress were assessed at baseline and after 3 and 6 weeks of intervention. Fecal samples were also collected at these same time points.

**Results:**

EpiCor fermentate led to a significant improvement of symptoms such as bloating/distension (*p* = 0.033 and *p* = 0.024 after 2 and 4 weeks of intervention, respectively), feeling of fullness (*p* = 0.004 and *p* = 0.023 after 2 and 4 weeks of intervention, respectively) and general daily scores (*p* = 0.046 after 2 weeks of intervention) in the moderate subgroup. A significant improvement in stool consistency was observed for the total population (*p* = 0.023 after 2 weeks of intervention) as well as for the severe subgroup (*p* = 0.046 after 2 weeks of intervention), and a nearly significant increase in stool frequency was detected for the total cohort (*p* = 0.083 and *p* = 0.090 after 2 and 4 weeks of intervention, respectively). These effects were accompanied by an improvement in constipation-associated quality of life and general perceived stress, particularly in the moderate subgroup. Members of the families Bacteroidaceae and Prevotellaceae, two groups of bacteria that have been previously reported to be deficient in constipated patients, were found to increase with EpiCor fermentate in the severe subgroup. In the moderate subgroup, a significant increase in *Akkermansia muciniphila* was observed.

**Conclusions:**

Despite the relatively low dose administered (500 mg/day), particularly when comparing to the high recommended doses for prebiotic fibers, EpiCor fermentate was able to modulate the composition of the gut microbiome, resulting in improvement of constipation-associated symptoms. Conversely, the reported increase in bowel movements may have altered the gut microbial community by increasing those groups of bacteria that are better adapted to a faster gastrointestinal transit time.

**Trial registration:**

NCT03051399 at ClinicalTrials.gov. Retrospectively registered. Registration date: 13 February 2017.

**Electronic supplementary material:**

The online version of this article (10.1186/s12906-017-1948-0) contains supplementary material, which is available to authorized users.

## Background

Functional constipation, also known as chronic idiopathic constipation (CIC), is a symptom-based gastrointestinal (GI) disorder without apparent organic abnormalities that occurs in otherwise healthy individuals [1]. However, constipation is a common complaint in clinical practice, and its prevalence ranges between 5% and 20% in the general population [2, 3], thereby representing a significant health care burden [4, 5]. In 2012, it was estimated to account for 3.2 million visits to medical centers in the United States [4, 5], with annual treatment costs of $1912–$7522 per patient [6]. In addition to economic costs, constipation greatly affects patients’ quality of life, having a significant impact on both mental and physical components [7, 8]. The efficacy of pro- and prebiotics in functional constipation has been recently reviewed, and it has been concluded that, in general, there is insufficient evidence to recommend probiotics for functional constipation, as considered trials are few, heterogeneous and poorly designed [9]. Although the evidence that prebiotic fibers (e.g. psyllium and inulin) have a positive effect on constipation is more substantial, and thus recommendations for their intake are stronger, the quality of evidence is still considered low. Many reasons may account for this, such as poor study design, heterogeneity of administered doses and duration of treatment [9]. However, it is our conviction that the inherent difficulties in studying constipation, due to a lack of objective markers (e.g. blood parameters), and the recognized placebo effect commonly observed throughout GI disorder trials are playing a major role [1, 10–13].

In most instances, it is challenging to show beneficial gut health effects within a target population of healthy individuals [14]. Therefore, most, if not all trials enrol specific patient groups in gut-health studies. In many examples, these relate to patients suffering from Irritable bowel syndrome (IBS) as defined by the Rome III criteria. IBS is a functional GI disorder characterized by chronic or recurrent abdominal pain or discomfort, mostly associated with defecation abnormalities (constipation alternating with diarrhea episodes) in the absence of a detectable organic or pathological cause. However, abdominal pain or discomfort occur both in healthy subjects and IBS patients, with frequency and/or severity of symptoms usually higher in IBS patients. Therefore, the Rome III diagnostic criteria distinguishes IBS from functional constipation, the latter being usually diagnosed after careful examination of patients’ history and in the absence of clear physiological abnormalities [1]. Despite some controversy [15], primary constipation (i.e., unrelated to medication use and/or neurological or systemic illness) is deemed idiopathic (unknown cause) and there are no specific markers that support diagnosis. Hence, patients’ history is the most determinant factor for correct diagnosis.

EpiCor, a dried fermentate made using yeast (*Saccharomyces cerevisiae*), while neither a probiotic nor a prebiotic fiber, has been shown to have immune-modulating properties in both human clinical trials [16–18] and in vitro [19–21]. Furthermore, a recent study using in vitro gut models has shown that EpiCor fermentate is selectively fermented by the intestinal microbiota in the colon, resulting in beneficial modulation of the intestinal microbiota and luminal environment [21]. The combination of these findings suggests that repeated intake of EpiCor fermentate can positively affect the intestinal environment in humans, thereby enhancing digestive comfort and ultimately contribute to improved immunity. Interestingly, the use of the Simulator of the Human Intestinal Microbial Ecosystem (SHIME®) has shown that EpiCor fermentate has prebiotic potential by increasing butyrate levels in the simulated colon and by stimulating Lactobacilli growth [21]. Butyrate, one of the main end-products from carbohydrate fermentation by the gut microbiota, is the main energy source for colonocytes, has recognized immunomodulatory activities and anti-cancer effects (reviewed in [22]). The *Lactobacillus* genus is also recognized as containing several health-enhancing species. Altogether, these results obtained in vitro indicate that long-term administration of EpiCor fermentate (up to 4 weeks) is able to modulate the intestinal environment and alter gut microbial composition, thereby suggesting an important prebiotic-like effect [21]. Importantly, there is evidence that GI motility and gut microbiota are clearly associated [23]. Experiments performed in humanized germ-free mice suggest that gut microbes modulate bowel movements, and changes in GI motility also modify the resident microbial population [24]. In addition, microbial metabolites, particularly short-chain fatty acids (SCFA), including butyrate, are also recognized as being essential for optimal ileal and colonic motor activity [23, 25]. Although most evidences have been obtained using animal models, the role of butyrate in altering GI transit is well described [26]. In addition, some studies have shown that altered microbiome composition is a common trait of both functional constipation and constipation-predominant IBS (C-IBS) [27–31].

Taking this collective body of evidence into account, we hypothesized that EpiCor fermentate, as a result of its prebiotic-like effect, may help improve bowel function and generally contribute to enhanced gut health. Therefore, this pilot study was intended to assess the effect of 6-week administration of EpiCor fermentate on GI symptoms and stool frequency/consistency in a population with moderate to severe symptoms of intestinal discomfort, and to determine if EpiCor treatment could lead to an improvement in quality of life. Additionally, fecal samples were collected at pre-defined intervals in order to investigate associated changes in gut microbiome composition.

## Methods

### Study design

**Fig. 1:**
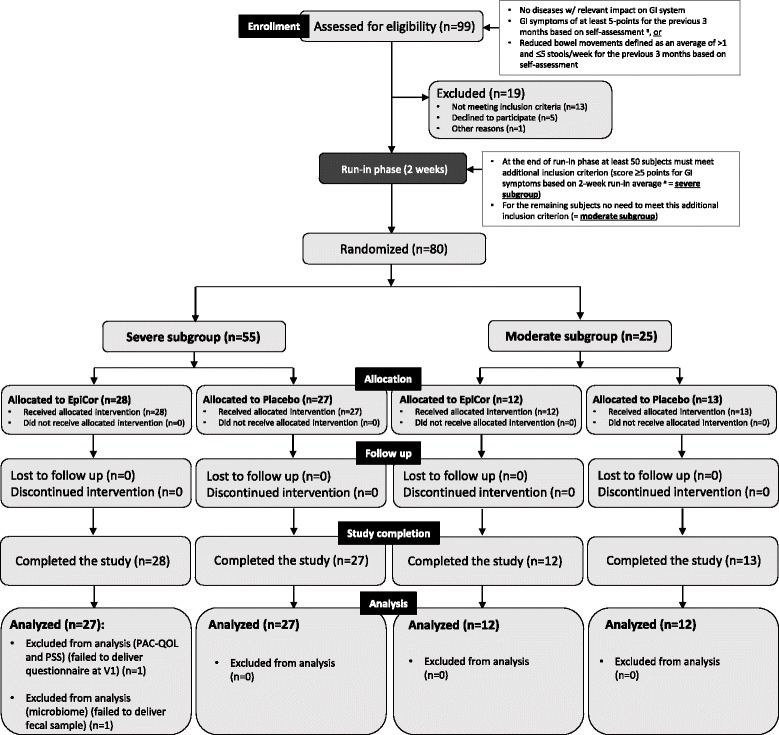
Schematic diagram of the study flow (based on CONSORT 2010 guidelines). Legend: GI, gastrointestinal; PAC-QOL, Patient Assessment of Constipation Quality of Life; PSS, Perceived Stress Scale. ^a^ Gastrointestinal symptoms were assessed by asking the volunteers to grade daily in the evening the average severity over the previous 24 h on a 5-point scale from 0 (not at all) to 4 (extremely) for the following 5 GI characteristics: bloating/distension, passage of gas, GI rumbling, feeling of fullness and abdominal discomfort

This exploratory study conformed to a mono-center, randomized, double-blind, placebo-controlled parallel design. Human male/female volunteers with moderate to severe symptoms of GI discomfort and constipation were screened for eligibility (Fig. 1). Eligible subjects, i.e., meeting the different inclusion and exclusion criteria (Table 1), were enrolled in a 2-week run-in phase so to provide baseline measurements of GI discomfort and stool frequency and consistency. The 2-week averages obtained for the GI symptoms diary were also used as an additional inclusion criteria in order to allocate the subjects to one of two subgroups according to the randomization scheme (see further below).

**Table 1:** List of inclusion and exclusion criteria for participation in the study

Inclusion criteria	Exclusion criteria
• Healthy volunteers without clinical diagnosed diseases with relevant impact on GI system or on visceral motility • GI symptoms of at least 5-points for the previous 3 months based on self-assessment using a 5-point scale questionnaire ^a^, or • Reduced bowel movements defined as an average of >1 and ≤ 5 stools per week for the previous 3 months based on self-assessment • Age ≥ 18 and ≤ 70 years • Male or female • No pregnancy in the 6 months prior to study • BMI: 18–35 kg/m^2^ • Stable body weight (± 5%) for at least 6 months • No weight reduction treatment during study period • Written consent to participate in the study • Able and willing to follow the study protocol	• History of severe GI/hepatic, hematological/immunologic, metabolic/nutritional disorders, endocrine disorders, celiac disease, type I diabetes mellitus, major surgery and/or laboratory assessments which might limit participation in or completion of study period • Use of medication, including vitamin supplementation, except oral contraceptives, within 14 days prior to first dosing. Some medication may be used, if it is considered not to influence GI function and motility • The use of any non-steroidal inflammatory drugs (NSAIDs) starting 14 days prior to first dosing is prohibited • Systemic antibiotics treatment within 60 days prior to first dosing • Intake of laxatives or anti-diarrheic drugs within 14 days prior to first dosing • Change of dietary habits within the 4 weeks prior to screening • Participants anticipating a change in lifestyle or physical activity levels during the study • Major abdominal surgery interfering with GI function • Known pregnancy or lactation • Dependence on illegal drugs or alcohol • Smoking within the last 3 months • Prohibited use of pro-, pre- or synbiotics from 30 days before first dosing and during the study period • Hepatitis C-, B- or HIV-positive • History of any major side effects towards intake of pro- or prebiotic supplements of any kind
Additional inclusion criterion	NA
• At the end of the run-in phase a score of ≥ 5-points for GI symptoms must be obtained at least for 50 subjects, based on the average calculated for the daily scores of the 2-week run-in period ^a^	NA

Legend: *BMI*, body mass index (calculated as weight in kg divided by length (m) squared); *GI*, gastrointestinal; *NA*, not applicable

^a^Gastrointestinal symptoms were assessed by asking the volunteers to grade daily in the evening the average severity over the previous 24 h on a 5-point scale from 0 (not at all) to 4 (extremely) for the following 5 GI characteristics: bloating/distension, passage of gas, GI rumbling, feeling of fullness and abdominal discomfort

This study was designed so that results would comply with all European Food Safety Authority (EFSA) requirements for scientific results that substantiate statements for ingredient efficacy. According to EFSA, claims related to the GI tract fall within the scope of ‘Function claims’ and ‘Claims on gastrointestinal discomfort’, and the recommendations for gut health studies are to use specific patient groups as study group, such as IBS and functional constipation. In agreement with the guidelines, GI discomfort may be measured by using validated subjective global symptom questionnaires [32]. Validated ‘quality of life questionnaires’ are also considered to provide supportive evidence for claims on GI discomfort. Therefore, we have made use of previously validated questionnaires in order to investigate the effects on symptoms of GI dysfunction, stool frequency/consistency and constipation-associated quality of life [14, 33] (see further below).

### Participants

Eighty healthy male/female volunteers between 18 and 70 years of age with reduced bowel movements and other symptoms of GI discomfort were enrolled via public notice board and phone call. All included participants finished the trial. Participants’ enrolment and study execution took place between July 2015 and January 2016 at the Drug Research Unit Ghent (D.R.U.G.) located at the Ghent University Hospital (Belgium), an independent study site performing clinical trials. The study was sponsored and coordinated by ProDigest BVBA (Belgium) and commissioned by Embria Health Sciences (USA). Before inclusion in the study, participants were medically examined for their physical health conditions and were assessed for the fulfillment of all inclusion and exclusion criteria (Table 1).

### Study product

EpiCor fermentate is the brand name for a substance consisting of a dried yeast fermentate made using *Saccharomyces cerevisiae* (produced by Embria Health Sciences, LLC, of Ankeny, Iowa, USA). It consists of various metabolites, including polyphenols, polysaccharides such as beta glucan, trace minerals, amino acids, and peptides. Within the USA, EpiCor fermentate has successfully completed the New Dietary Ingredient Notification process with the US Food and Drug Administration (FDA) and has been determined to be a Generally Recognized as Safe ingredient by an independent panel of safety experts. Table 2 shows the nutritional information for bulk dried fermentate.

**Table 2:** Nutritional details of three lots of EpiCor®

Nutritional details		Lot number		
		0064–230,714	0065–180,814	0066–020914
Calories	kcal/100 g	330	329	327
Carbohydrates	%	50.49	47.92	45.06
Sugars	%	<0.35	<0.35	<0.35
Total Fat	%	1.78	1.77	1.82
Cholesterol	mg/100 g	<0.8	<0.8	<0.8

So far, all published human studies on EpiCor fermentate have used a daily dose of 500 mg for adults [16–18, 34]. Moreover, this is the commercially recommended daily dosage, and so was also the dose used here. The placebo used in this study was Globe maltodextrin 10 (CPIngredientes, Mexico). This commercially available product is a mixture of dextrose, maltose, oligo and polysaccharides obtained by partial enzymatic hydrolysis of corn starch. Maltodextrin is the most commonly used placebo in dietary studies evaluating gut microbiota and intestinal well-being. It is easily digested and rapidly absorbed as glucose and has no anticipated effect on colonic fermentation. Both products were provided in capsules. The capsules were Coni-Snap®, two-piece hard gelatin capsules (Capsugel, Mexico). Blinding was ensured by the fact that both capsules were opaque and had an identical appearance and were packed in identical bottles by Embria Health Sciences and were labeled as ‘A’ or ‘B’ before shipment to ProDigest. A ProDigest staff member not participating in study design, sample processing or data analysis, randomized the participants, labeled all bottles and assigned them to each subject in accordance to the randomization list. In this manner the corresponding product was not known by either the sponsor members managing the study or the D.R.U.G. unit.

### Randomization scheme

As mentioned above, an additional inclusion criterion was defined after run-in phase in order to distinguish those subjects that have more severe symptoms from those who report to have more moderate symptoms. To do this, the daily questionnaires completed during the 2-week run-in phase were analyzed at the end of this period. This allowed us to obtain a more accurate evaluation of the effective GI symptoms perceived by the participants, as opposed to the single assessment performed during enrolment. To ensure that sufficient individuals with higher (severe) GI symptoms’ scores would be included, the threshold for successful inclusion in the study was set to have at least 50 subjects reporting an average score ≥ 5 for GI discomfort based on the 5-item GI symptoms questionnaire (see further below) which was recorded daily during run-in. The remaining 30 subjects did not have to meet this additional criterion. To ensure that within the two subgroups (severe and moderate) there would be an even number of subjects allocated to both trial arms (EpiCor fermentate or placebo), randomization was stratified [35] for symptom severity as follows: after screening and subsequent inclusion, each subject was assigned a unique subject identifier. Subjects were randomly assigned to one of the two testing conditions: 1) placebo (500 mg/day, single serving, maltodextrin) or 2) EpiCor fermentate (500 mg/day, single serving). In total, 80 subjects completed the study (40 subjects in each trial arm). The randomization scheme was generated by using the Web site Randomization.com (http://randomization.com). Randomization was done by using a permutated block design (blocks of 2, 4 and 6) in a stratified manner in order to allocate an even number of individuals from both trial arms to both subgroups. For that, two randomization lists were generated: one for those subjects (minimum 50) that met the additional inclusion criterion after run-in phase (GI symptoms average score ≥5) – here designated severe subgroup (subjects received a unique identifier number)**.** A second list was generated for the remaining subjects who did not meet this after run-in inclusion criterion (GI symptoms <5) – here designated moderate subgroup (subjects received a unique identifier number non-overlapping with the severe list). The individual identifiers were used to prepare labels so to assign to each subject the bottles containing either EpiCor or placebo according to the randomization scheme (‘A’ or ‘B’) for the entire study.

**Fig. 2:**
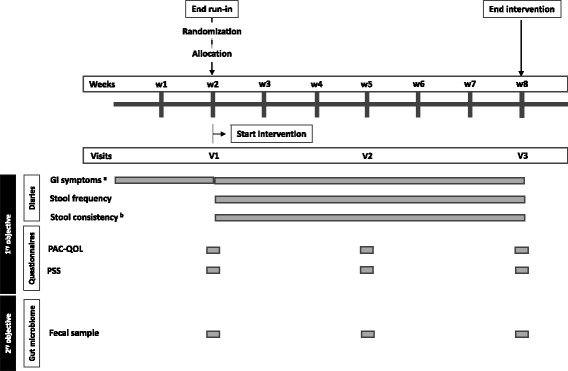
Schematic diagram of primary and secondary objectives and instruments used for data/sample collection. Legend: GI, gastrointestinal; PAC-QOL, Patient Assessment of Constipation Quality of Life; PSS, Perceived Stress Scale. ^a^ Gastrointestinal symptoms were assessed by asking the volunteers to grade daily in the evening the average severity over the previous 24 h on a 5-point scale from 0 (not at all) to 4 (extremely) for the following 5 GI characteristics: bloating/distension, passage of gas, GI rumbling, feeling of fullness and abdominal discomfort. ^b^ Stool consistency was recorded according to the Bristol Stool Form Scale

After receiving the GI symptoms diary recorded during run-in, an average total score was calculated for the two-week period and the subjects allocated to the corresponding randomization list. At the end of the study, the number of subjects within the severe subgroup was 55 and within the moderate subgroup 25 (Fig. 1). After randomization, a baseline visit was scheduled and samples were collected for baseline parameters (visit 1) (Fig. 2). Subjects were also asked to fill in the questionnaires Patient Assessment of Constipation Quality of Life (PAC-QOL) and Perceived Stress Scale (PSS) (see further below) in order to assess their baseline symptoms for each questionnaire. They have also received the corresponding testing product (either EpiCor or placebo) and initiated the 6-week intervention trial (500 mg/day, single dose). Two more visits were scheduled: after 3 and 6 weeks (visits 2 and 3, respectively). At each visit, fecal samples were collected and questionnaires filled in. Throughout the entire study, the subjects also filled in a diary, where they could record daily their GI symptoms, stool frequency and stool consistency (Fig. 2). The baseline instrument for the intervention diary was the run-in diary that was used for allocation. The randomization scheme was done by a ProDigest staff member not involved in the study. All participants, principal investigators and staff members involved in the study at both D.R.U.G. and ProDigest sites were blinded. Unblinding only occurred after completion of data analysis. The key for identifying the products in case of adverse events was kept sealed in an envelope at D.R.U.G. and ProDigest until unblinding. No adverse events were recorded.

### Primary objective

The primary objective of this pilot study was to study the effect of long-term administration of EpiCor on digestive comfort and constipation-associated quality of life. To assess this, the volunteers were asked to document daily (during the 2-week run-in phase and 6-week intervention phase) their GI symptoms as well as the frequency and consistency of their stools. For assessment of GI symptoms, the volunteers were asked to grade daily in the evening the average severity over the previous 24 h on a 5-point scale from 0 (absent) to 4 (very severe) the following five items: Bloating/Distension, Passage of gas, GI Rumbling, Feeling of fullness and Abdominal discomfort (this instrument has been described and used by Buchwald-Werner and colleagues (2014) in a similar study [14]). A Daily Total Score (DTS) was also calculated by summing all items recorded each day. A lower score is concomitant with lower severity of symptoms. Stool frequency and consistency were also recorded daily by using the Bristol Stool Form Scale [36]. This comprises seven types of stool: type 1 (separate hard lumps); type 2 (sausage shape lumpy); type 3 (sausage with cracks); type 4 (sausage but soft and smooth); type 5 (soft lobs); type 6 (fluffy and mushy) and type 7 (liquid). Types 1, 2 and 3 are associated with hard or impacted stools (linked with dysbacteriosis and chronic constipation); types 4 and 5 are considered normal or optimal; type 6 is considered subnormal or suboptimal and type 7 is associated with diarrhea. Both instruments were subject to a 2-week period evaluation: average of weeks 1 and 2 of run-in (=T1; baseline); average of weeks 1 and 2 of intervention (=T2); average of weeks 3 and 4 of intervention (=T3) and average of weeks 5 and 6 of intervention (=T4). The GI symptoms diary reported during run-in was also used as an instrument to allocate subjects to the corresponding subgroups and according to the randomization list (EpiCor severe and EpiCor moderate, placebo severe and placebo moderate) (see Figs. 1 and 2).

Constipation-associated quality of life was evaluated by using the Patient Assessment of Constipation Quality of Life (PAC-QOL) questionnaire [33] (Janssen Global Services, LLC, USA; MAPI Research Trust, France), which has been validated in a patient population with history of chronic constipation. The PAC-QOL provides information about the special distraction of daily life and general well-being of volunteers because of constipation [37]. Volunteers were asked to fill in this questionnaire retrospectively at baseline, middle and end of intervention (visits 1, 2 and 3, respectively) (Fig. 2). The PAC-QOL questionnaire is a 28-item self-reporting instrument divided in four domains: Physical Discomfort, Psychosocial Discomfort, Worries and Concerns and Satisfaction. A 5-point scale from 0 (none of the time) to 4 (all of the time) was used to assess the severity of the different symptoms. A final Instrument Total Score (ITS) was also used by calculating the mean of the 28 items at each visit. A lower score is concomitant with a better quality of life.

It is known that psychosocial factors, such as daily stress may alter gut physiology leading to ileum contractions and consequently to GI discomfort [38]. Therefore, subjects were asked to scale their stress levels in the Perceived Stress Scale (PSS) questionnaire [39]. This is the most widely used psychological instrument for measuring the perception of stress (not constipation related) [39]. Volunteers were asked to fill in this questionnaire retrospectively at the same days as for PAC-QOL. The PSS is a 10-item self-reporting instrument with a 5-point scale from 0 (never) to 4 (very often). A final Instrument Total Score (ITS) was calculated by summing all items recorded at each visit. A lower score is concomitant with lower stress. Although the first intent was to rule out the role of stress from the study, the results obtained with this instrument paralleled the ones obtained for the PAC-QOL. Thus, despite the obligation of not altering objectives after data analysis, given the fact that improvement of quality of life and decrease in stress levels are somewhat related, it is our conviction that these instruments can be regarded as being complementary.

### Secondary objective

Given established links between constipation and gut microbiome dysbiosis [23, 24, 27–31, 40], the secondary objective of this study was to assess the effect of EpiCor fermentate on gut microbial composition. For that, subjects were asked to collect fecal samples at visits 1 (baseline), 2 and 3 (3 and 6 weeks after intervention, respectively) (Fig. 2). Participants were also instructed to store the sample container in the freezer until delivery. Total DNA was extracted using the Fast-Prep24 instrument (MP-Biologicals), as previously described [41]. Briefly, 100 mg of fecal sample were resuspended in Tris/HCl (100 mM, pH 8.0) supplemented with 100 mM Ethylenediaminetetraacetic acid (EDTA), 100 mM sodium chloride (NaCl), 1% (*w*/*v*) polyvinylpyrrolidone and 2% (wt/vol) sodium dodecyl Sulphate (SDS) and mechanically disrupted. Bacterial cells were lysed in a Fast Prep-24 instrument (40 s., 6.0 m/s.). Samples were then centrifuged at 20,800 *g* for 5 min and the supernatant washed with one volume phenol/chloroform/isoamylalcohol (25:24:1), followed by another centrifugation step. Then, the aqueous phase was washed with one volume chloroform. After centrifugation, nucleic acids (aqueous phase) were precipitated with one volume of ice-cold isopropanol and 1:10 volume of 3.0 M sodium acetate. The DNA was resuspended in 100 μl sterile TE-buffer (10 mM Tris-HCl, 1 mM EDTA, pH 8.0). Before proceeding with PCR amplification, a cleaning step was performed with the OneStep™ PCR Inhibitor Removal Kit (Zymo Research, USA). DNA quality and quantity were analyzed by electrophoresis on a 1.2% (*w*/*v*) agarose gel and by determination of the absorbance at 260 and 280 nm. Assessment of qualitative changes in the general microbiota structure and profiles were done by Illumina® sequencing, a technique involving the amplification of a hypervariable region (V5-V6 region of the 16S ribosomal RNA) of bacterial DNA and sequencing of the amplified region. This region was amplified using previously reported primers [42].

### Statistical analysis

For the primary objectives (diaries and questionnaires), a linear mixed model analysis was used to determine the longitudinal effects of intervention. This model corrects for baseline differences and includes all patients, even those who drop out during intervention (note however that there were no dropouts). Group, time and group-by-time were included as fixed factors. An unstructured covariance structure for repeated measures was considered. As a result, random effects were redundant. No multiple imputation method was required, since group and time did not have any missing data and a likelihood-based approach for missing outcome data was used. All analyses were performed using IBM SPSS Statistics for Windows (Version 23.0, Armonk, NY, USA). The estimated effects (differences between EpiCor fermentate and placebo) ± 95% 2-sided confidence interval (CI) were plotted in forest plots. Differences between EpiCor fermentate and placebo for the total cohort as well as for both subgroups (severe and moderate) were calculated separately. In addition, statistical significant differences ‘within’ groups were calculated by using one-way repeated measures analysis of variance (ANOVA) in GraphPad Prism (v7.00 for Windows, GraphPad Software, La Jolla California USA, www.graphpad.com). A *p-*value ≤0.05 was considered statistically significant, although nearly significant *p*-values (*p* < 0.1) are also indicated if deemed relevant.

Concerning the Illumina® sequencing data (secondary objective), the V5-V6 region of the 16S rRNA gene was amplified using previously reported primers [42]. Libraries were prepared by pooling equimolar ratios of amplicons, using 200 ng of each sample, tagged with a unique barcode [43]. Resulting libraries were sequenced on a MiSeq (Illumina, Hayward, CA, USA) paired and joined, but only forward reads were selected for the final analysis (140 nucleotides). A quality filter program that runs a sliding window of 10% of the read length, and calculates the local average score based on the Phred quality score of the FASTQ file, was used to trim the 3′-ends of the reads that fell below a quality score of 10. Reads with an N character in their sequence, mismatches within the primers and barcodes or more than 8 homopolymers stretches were discarded. Following primer sequences trimming, sequences were separated based on their barcodes. The number of representative phylotypes was generated using the Uclust algorithm on USEARCH [44] by clustering at 97% similarity (1 mismatch), with a confidence level of at least 80, with Cyanobacteria, Eukaryota, and Archaea lineages removed. Filtered database contained only phylotypes present in at least: 1) one sample at an abundance higher than 1%, 2) in 2% of samples at a relative abundance above 0.1%, and 3) in 5% of the samples at any abundance level [43]. Sequence composition was compared using the RDP Classifier tool [45] and SILVA database [46]. Based on Pareto-Lorenz evenness curves [47] adapted for microbial diversity [48], which plot species cumulative abundance, a selection of the 400 most abundant operational taxonomic units (OTUs) was done so to obtain a representative overview of microbial community changes. Relative abundances of these OTUs were further processed at phylum, family and genus levels. In total, these 400 OTUs were classified into 6 phyla, 30 families and 58 annotated genera. To evaluate differences across time within the two treatment groups at both family and genus level, two-way repeated measures ANOVA with Dunnett’s multiple comparison’s test against V1 was performed in GraphPad Prism (v7.00 for Windows). A *p-*value ≤0.05 was considered statistically significant, although nearly significant *p*-values (*p* < 0.1) are also indicated. Boxplots were also done in GraphPad Prism according to the Tukey method. Data analysis was performed for the total cohort and the two subgroups (severe and moderate) separately. To highlight those taxa that mostly explain the differences between placebo and EpiCor fermentate treatments, a joint principal component analysis (PCA) / correlation biplot was performed by using the relative fold-changes (V2/V1 and V3/V1) calculated for all three cohorts with the help of Analyse-it for Microsoft Excel 4.51 software. This explorative method allows analyzing the association between variables (depicted as vectors) and observations (depicted as points) by projecting them into the same two-dimensional space. Logarithmic (Log2) transformed fold-changes were also imputed into MeV 4.9.0 (Multiexperiment Viewer) software so to perform a hierarchical clustering (HCL) analysis in the form of heatmaps [49]. The K-means clustering (KMC) method was used by setting the number of clusters to one and performing 10,000 iterations. A Pearson correlation distance metric was used to build the hierarchical clustered tree. Both taxa and observations were clustered. The resulting heatmap showed approximately the presence of 7 major clusters. This study report conforms to the CONSORT 2010 guidelines.

## Results

The distribution of patients per trial arm and within each subgroup is depicted in Table 3. In all groups, the age range of participants was approximately between 20 and 69 years, and the median around 50 years of age. As it is known that women suffer more from gastrointestinal discomfort and constipation [14, 50, 51], more women than men were included in this study. Because randomization was stratified for symptom severity, an even number of subjects was allocated to either treatment arm within each subgroup. This also ensured that there were no substantial differences in GI symptoms daily total scores (DTS) at baseline between EpiCor fermentate and placebo within each subgroup (severe and moderate) (Table 3).

**Table 3:** Subjects allocation per trial arm and subgroup and baseline characteristics

			Gender		Age		BMI (kg/m^2^)		GI symp. DTS ^a^
Cohort	Treatment	*n*	Males (*n*)	Females (*n*)	Range	Median	Range	Median	Mean (± SEM)
Total cohort (*n* = 80)	EpiCor	40	7	33	20–69	50	18–33	24	7.20 ± 0.56
	Placebo	40	6	34	21–65	45	18–35	24	6.56 ± 0.46
Severe (*n* = 55)	EpiCor	28	5	23	24–66	48	18–33	24	8.68 ± 0.60
	Placebo	27	3	24	21–65	44	18–33	24	8.08 ± 0.42
Moderate (*n* = 25)	EpiCor	12	2	10	20–69	57	19–33	23	3.74 ± 0.31
	Placebo	13	3	10	23–63	52	20–35	22	3.41 ± 0.32

Legend: *BMI*, body mass index; *GI*, gastrointestinal

^a^Average of daily total scores (DTS) obtained after 2-week run-in on GI symptoms. This score was used to allocate subjects within the two subgroups (severe: GI symptoms ≥ 5 and moderate: GI symptoms <5)

### Primary objective

**Fig. 3:**
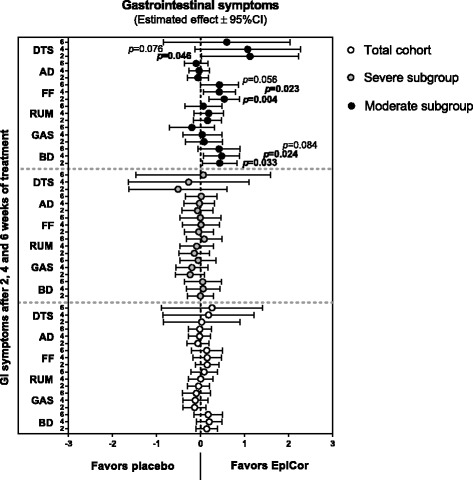
Mean differences between EpiCor and placebo on gastrointestinal symptoms on the three cohorts. Legend: The enlisted gastrointestinal symptoms are BD (bloating/distension), GAS (passage of gas), RUM (GI rumbling), FF (feeling of fullness) and AD (abdominal discomfort). A daily total score (DTS) calculated as the sum of all items recorded each day is also shown. A linear mixed model analysis that takes into account the differences between groups at baseline was used (*p*-values <0.05 are depicted in bold text; *p*-values <0.1 are depicted in regular text)

**Fig. 4:**
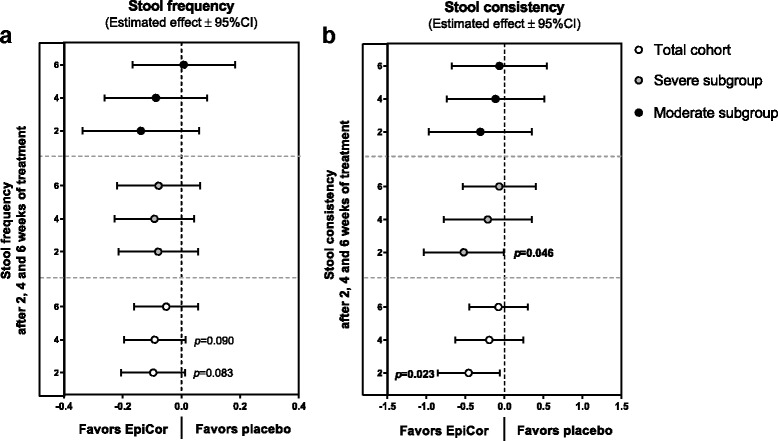
Mean differences between EpiCor and placebo on stool frequency (**a**) and consistency (**b**) on the three cohorts. Legend: A linear mixed model analysis that takes into account the differences between groups at baseline was used (*p*-values <0.05 are depicted in bold text; *p*-values <0.1 are depicted in regular text)

Two-week-interval averages obtained from the daily reported GI symptoms were calculated: average of weeks 1 and 2 of run-in (=T1; baseline); average of weeks 1 and 2 of intervention (=T2); average of weeks 3 and 4 of intervention (=T3) and average of weeks 5 and 6 of intervention (=T4) (Additional file: 1). A one-way repeated measures ANOVA was used to estimate if changes over time were significant within each treatment group. In this study, a noticeable placebo effect was observed for many endpoints, a result which is not unexpected for gut health-related trials [1, 10–13] (Additional file: 1). The placebo effect was particularly evident in the severe subgroup. In this subgroup no significant differences between EpiCor fermentate and placebo were found (see also Fig. 3). However, ‘between groups’ analysis clearly showed that EpiCor fermentate had significant positive effects on bloating/distension (*p* = 0.033 and *p* = 0.024 after 2 and 4 weeks of treatment, respectively), feeling of fullness (*p* = 0.004 and *p* = 0.023 after 2 and 4 weeks of treatment, respectively) and general GI discomfort (as evaluated from the daily total scores; *p* = 0.046 after 2 weeks of treatment) on those subjects reporting milder symptoms (moderate subgroup) (Fig. 3). Despite the placebo effect noticed on GI symptoms, a nearly significant improvement on stool frequency (*p* = 0.083 and *p* = 0.090 after 2 and 4 weeks of treatment, respectively) and a significant improvement on stool consistency (*p* = 0.023 after 2 weeks of treatment) was observed for the EpiCor-treated total cohort (Fig. 4). The averages calculated for all time points and the statistical significant differences ‘within groups’ over time can be seen in Additional file: 2. Here it is also evident that a significant improvement of stool consistency is observed over time in the EpiCor-treated total cohort (*p* = 0.037) as opposed to placebo (*p* = 0.535). This is also observed within the severe (EpiCor, *p* = 0.044; placebo, *p* = 0.424) and moderate (EpiCor, *p* = 0.031; placebo, *p* = 0.425) subgroups.

**Fig. 5:**
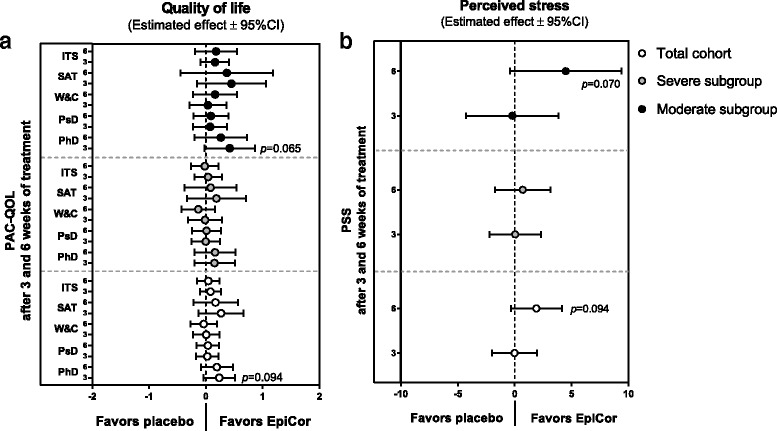
Mean differences between EpiCor and placebo on constipation-associated quality of life (**a**) and perceived stress (**b**) on the three cohorts. Legend: The enlisted Patient Assessment of Constipation Quality of Life (PAC-QOL) items are PhD (physical discomfort), PsD (psychosocial discomfort), W&C (worries and concerns) and SAT (satisfaction). An instrument total score (ITS) calculated as the average of all 28 items recorded at each visit is also shown. PSS relates to the Perceived Stress Scale instrument. A linear mixed model analysis that takes into account the differences between groups at baseline was used (no *p*-values <0.05 were found; *p*-values <0.1 are depicted in regular text)

Finally, the impact of constipation on quality of life and general perceived stress have been assessed (Additional file: 3 and Fig. 5). For the PAC-QOL instrument, a pronounced placebo effect was also noted, as subjects reported an improvement in their quality of life in both treatment groups (Additional file: 3). However, again, the moderate subgroup was less affected by this placebo effect, and a significant improvement of items such as physical discomfort (*p* = 0.017), psychosocial discomfort (*p* = 0.027) and satisfaction (*p* = 0.013) was reported within the EpiCor-treated group, as opposed to the placebo group (*p* = 0.435, *p* = 0.129 and *p* = 0.166, for physical discomfort, psychosocial discomfort and satisfaction, respectively) (Additional file: 3). In spite of the fact that the differences between EpiCor fermentate and placebo did not reach significance, it is clear a tendency for improvement in the EpiCor-treated group in items such as physical discomfort (which reached nearly significant levels on the total cohort and moderate subgroup) and satisfaction (Fig. 5a). Regarding general stress levels (PSS instrument) a significant decrease in stress levels over time was reported within the EpiCor-treated group (*p* = 0.016 and *p* = 0.044 for the total cohort and severe subgroup, respectively) in contrast to the placebo-treated group (*p* = 0.846 and *p* = 0.555 for the total cohort and severe subgroup, respectively) (Additional file: 3). The differences ‘between groups’ were also quite pronounced, despite lack of significance (Fig. 5b). Here, a nearly significant reduction in stress levels was observed for EpiCor-treated total cohort (*p* = 0.094) and moderate subgroup (*p* = 0.070).

### Secondary objective

From literature it is clear that constipation is associated with a dysbiotic gut microbial community [23, 24, 27–31, 40]. Thus, in order to investigate whether EpiCor consumption altered the gut microbial composition, fecal samples were collected at visits 1 (baseline), 2 and 3. DNA was extracted and the hypervariable region (V5-V6) of the bacterial 16S was amplified and sequenced. From the approximately 100,000 OTUs obtained upon sequencing, 400 were selected based on their relative abundance (Additional file: 4). Based on a Pareto-Lorenz curve [47] adapted for microbial biodiversity [48], which describes the unequal distribution of bacterial species among the entire data set, this subset of OTUs was found to account for more than 90% of the relative abundance, and so was the one chosen to be used for further analysis.

**Fig. 6:**
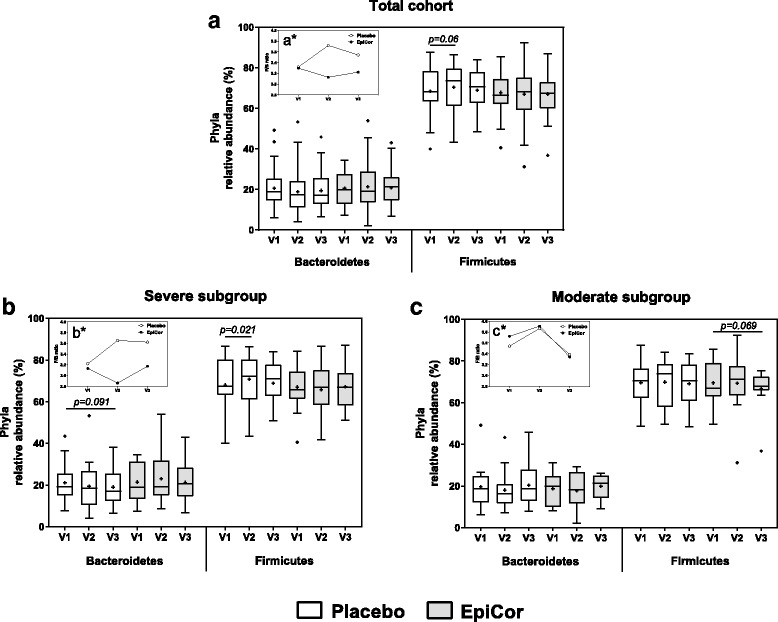
Bacteroidetes and Firmicutes relative abundances within the total cohort (**a**) and the two subgroups, severe (**b**) and moderate (**c**) that have been treated either with placebo or EpiCor. Each box represents median (50th percentile) and interquartile range (25th and 75th percentiles). The symbol (+) represents the mean. The outliers are indicated as dots (Tukey method). Significant (*p* < 0.05) and nearly significant (*p* < 0.1) *p*-values are also indicated within the boxplots (two-way repeated measures ANOVA with Dunnett’s multiple comparison’s test against V1). The inner plots (a*, b* and c*) correspond to the calculated Firmicutes/Bacteroidetes (F/B) ratio at each visit within the two treatment groups. Legend: V1, V2 and V3 correspond to visit 1 (baseline), visit 2 (3-weeks after treatment) and visit 3 (6-weeks after treatment), respectively

**Fig. 7:**
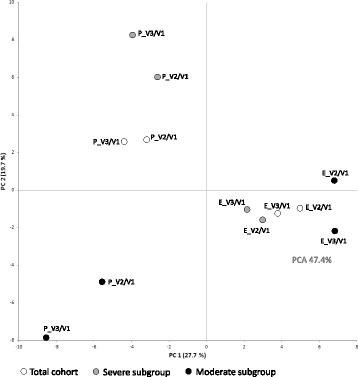
Principal component analysis (47.4%) of the relative fold-changes calculated for all taxonomic groups (family and genus levels). Each dot represents a treated group (either EpiCor or placebo) for all three cohorts (total cohort, severe and moderate). The first component (PC1) accounts for nearly 28% of the variance, and the second component (PC2) for nearly 20%. legend: E, Epicor; P, placebo; V, visit

**Fig. 8:**
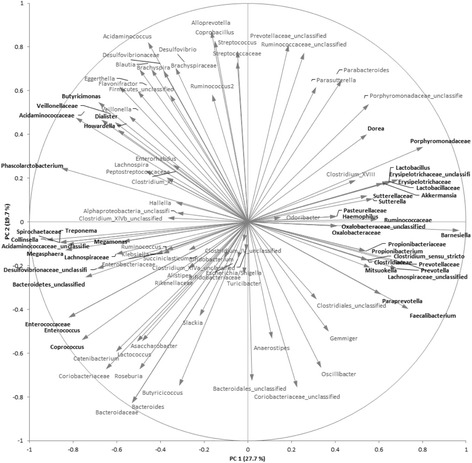
Variables (taxa) correlation plot. Each vector represents a taxonomic group (family or genus levels). Shorter vectors only slightly contribute for differentiation between groups. Longer vectors have a bigger weight in groups’ differentiation. Those variables that mostly contribute the first component (PC1) are indicated in bold text

**Fig. 9:**
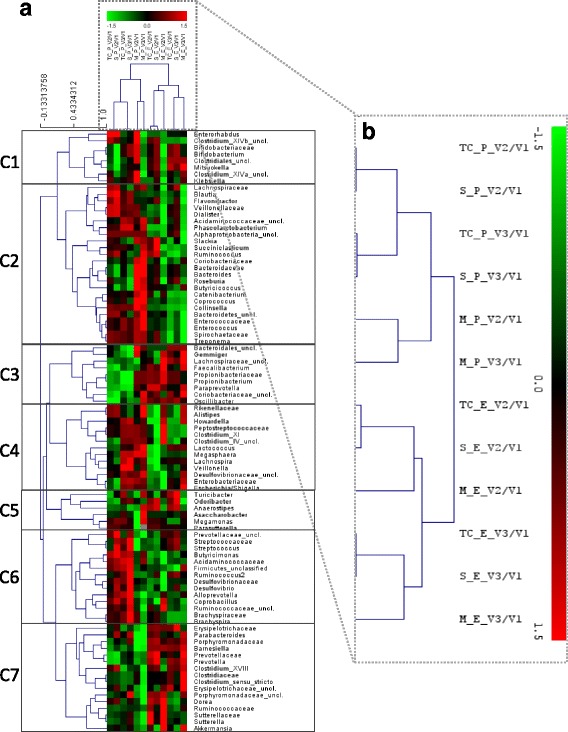
Hierarchical clustering heatmap of the Log2 relative fold-changes calculated for all taxonomic groups (family and genus levels). (**a**) Both taxa and treatment groups were subject to HCL analysis. (**b**) Shows a detail of the results for the groups’ HCL. The KMC analysis roughly revealed the presence of 7 major clusters (C1-C7) based on similarly of taxa relative fold-changes. Legend: C, cluster; E, Epicor; M, moderate subgroup, P, placebo; S, severe subgroup; TC, total cohort; V, visit

Within the gut, bacteria can be classified into one of six phyla: Actinobacteria, Bacteroidetes, Firmicutes, Proteobacteria, Verrucomicrobia and Spirochaetae, with the first four being the most dominant. At first glance, the relative abundance of these phyla does not seem to substantially change over time within the three cohorts (total, severe and moderate) (Additional file: 5). However, after a careful look at the two most abundant phyla, Bacteroidetes and Firmicutes, it is clear that within the EpiCor-treated group the Firmicutes/Bacteroidetes (F/B) ratio decreases over time, whereas in the placebo-treated group the F/B ratio increases (Fig. 6a and a*). This finding was mainly due to changes seen in the severe subgroup (Fig. 6b and b*) and was not observed within the moderate subgroup (Fig. 6c and c*). This suggests that within the severe subgroup, the relative abundance of Bacteroidetes is increasing after EpiCor fermentate consumption, in contrast to the placebo-treated severe subgroup. However, at phylum level, changes are usually mild, unless in the presence of a serious bowel-related disease, and are also generally less informative. Therefore, in order to better understand, within each phyla, which are the main groups of bacteria changing over time after placebo and EpiCor fermentate consumption, the fold-changes (relatively to baseline) were calculated for each bacterial taxonomic level (family and genus) (Additional files: 6, 7 and 8). It must stressed that although subjects were asked not to drastically alter their dietary habits throughout the study, diet was not monitored, and so it is difficult to account for diet-induced biases. Moreover, subjects enrolling in a study investigating the effects of dietary supplements may alter their dietary habits even if in an unconscious manner, which may confound results and lead to placebo effects. This could explain the fact that also within the placebo-treated group statistical significant differences were found for some taxonomic groups. To better evaluate which taxa are contributing to discriminate placebo from EpiCor within the three cohorts, a joint PCA / Correlation biplot was done (Figs. 7 and 8). The most obvious observation is the fact that the first component separates the placebo-treated groups from the EpiCor-treated groups (Fig. 7). The variables that mostly contribute to this separation are indicated in the correlation plot in bold text (Fig. 8). For example, some groups of bacteria are increasing upon EpiCor intake (at least in one of the cohorts), thereby contributing to Placebo vs. EpiCor differentiation in the first component. The groups showing the highest weight (as highlighted by longer vectors) are for instance the families Porphyromonadaceae, Lactobacillaceae and Prevotellaceae and the genera *Barnesiella*, *Prevotella* and *Akkermansia* (Fig. 8). The second important observation is that the total cohort is positioned between the severe and moderate subgroups, a fact that supports subgroup analysis. The subjects allocated to either subgroup were not only reporting different degrees of GI symptoms (Table 3), but were also found to differ in their microbial community composition at baseline, clearly indicating a higher degree of gut microbial dysbiosis for the severe subgroup (results not shown). Interestingly, after hierarchical clustering (HCL) analysis, it became apparent that time is also an important factor playing a role (Fig. 9). The EpiCor-treated groups cluster together per visit rather than per subgroup, thus suggesting relatively similar changes in microbial composition at visit 2 and visit 3. This analysis also revealed the presence of seven major clusters (C1-C7) with similar expression that somewhat overlap the results of the PCA/Correlation plot. For instance, in general, taxa belonging to the clusters C3 and C7 show an increase in the EpiCor-treated groups, whereas in the placebo there is a decrease or no change (e.g. *Propionibacterium*, *Paraprevotella* and *Oscillibacter* within C3 and *Barnesiella*, *Prevotella* and *Akkermansia* within C7). The relative increase in Bacteroidetes observed within the severe subgroup (Fig. 6) seems to be mainly attributed to an increase in members of the families Bacteroidaceae, Porphyromonadaceae and Prevotellaceae, with significant increases for the genera *Bacteroides* (*p* = 0.015 and *p* = 0.027 at V2 and V3, respectively) and *Prevotella* (*p* = 0.039 at V2) (Additional file: 7). Despite a lack of significance, a noticeable relative increase was also observed for the genera *Barnesiella* and *Odoribacter* (family Porphyromonadaceae) within the severe subgroup that received EpiCor. The relative decrease in Firmicutes is mostly apparent in cluster C2 (Fig. 9), as most members of this cluster (15 out of 24) belong to this phylum and, in general, they are decreasing upon EpiCor fermentate intake. Nevertheless, *Anaerostipes* (phylum Firmicutes) is significantly increasing in the EpiCor-treated group (*p* = 0.001 and *p* = 0.003 at V2 and V3, respectively) (Additional file: 7). Within the moderate subgroup changes are somewhat less evident, which may reflect the lower number of subjects included. Notably, however, within the moderate subgroup there is a significant relative increase of *Akkermansia muciniphila* (*p* = 0.0001 and *p* = 0.036 at V2 and V3, respectively), and a significant relative decrease in *Blautia* (*p* = 0.023 and *p* = 0.001 at V2 and V3, respectively) and *Roseburia* (*p* = 0.002 at V2), effects that were not observed within the severe subgroup (Additional files: 7 and 8).

## Discussion

The aim of this pilot study was to investigate the effect of EpiCor fermentate on GI discomfort by treating a population with symptoms of constipation and compare it to placebo in a parallel study. Because functional constipation is usually diagnosed based on patients’ history and therefore prone to being subjective [1], this study defined clear inclusion criteria. Therefore, patients were only included if they have reported (based on self-assessment using a validated questionnaire over the previous 3 months) GI symptoms of at least 5 points [14], or reduced bowel movements defined as an average > 1 and ≤5 stools per week. Following initial inclusion, subjects initiated a run-in phase, after which an additional inclusion criterion was established: at least 50 subjects should report an average score ≥ 5 for GI discomfort calculated based on the 5-item GI symptoms diary filled in during the 2-week run-in. This allowed us to recruit individuals that actually suffered from constipation, and to stratify the study population into two subgroups: one larger group with marked symptoms of GI discomfort (severe subgroup, *n* = 55) and a second group having milder symptoms (moderate subgroup, *n* = 25). This also allowed us to investigate treatment efficacy on two subgroups of patients suffering from different degrees of GI discomfort, as it is possible that efficacy differs according to symptom severity [10, 13].

The two subgroups of patients were shown to differ in terms of symptom severity, stool frequency and consistency and quality of life parameters at baseline, with the severe subgroup reporting more severe symptoms of GI discomfort (Table 3), fewer bowel movements and poorer quality of life, as expected (results not shown). In addition, they were also found to possess a more dysbiotic gut microbial community when compared to the moderate subgroup (results not shown). Similar results have been found by others both in the adult population [27, 30] and in children suffering from constipation [28, 29, 31, 40]. These and our results therefore suggest that constipation is associated with a dysfunctional gut microbiome, and evidences support that gut motility can be managed by intervening at the level of the gut microbial community [24, 52, 53]. Importantly, others have shown that relief of constipation by synthetic laxatives such as Bisacodyl tend to normalize and restore gut microbial composition, thus suggesting that dysbiosis is secondary, rather than a cause of constipation [30].

### Improvement of symptoms

The generally accepted outcomes for trials on functional gastrointestinal disorders (FGIDs), such as functional constipation, are those that reflect the patient’s symptoms that are relevant to the disorder and/or have an impact on quality of life [13]. Therefore, we have made use of validated instruments to assess the effects of EpiCor fermentate on GI discomfort, bowel movements and quality of life [14, 33, 36, 37, 39].

For that reason, the primary objective of this pilot study was to assess the effect of long-term administration of EpiCor fermentate on bowel function and gastrointestinal well-being by means of validated questionnaires. Despite a clear placebo effect within the severe subgroup, in the moderate subgroup EpiCor fermentate had a positive effect over time on five out of six GI symptoms domains. In the moderate subgroup significant effects compared to placebo were reached for bloating/distention, feeling of fullness and daily total score (Fig. 3). The underlying reason behind the strong placebo effect within the severe subgroup can only be speculated, but it is known that placebo response on FGIDs trials is particularly high, making it rather difficult to show superiority of a new treatment over placebo [10–13]. In that respect, individuals experiencing symptoms that are more pronounced may be more prone to subjective feelings of improvement, irrespective of the physiological effect of the treatment. This is commonly referred to as ‘regression to mean’, i.e., subjects experiencing severe intestinal discomfort will inevitably improve [10]. Interestingly, placebo effects were less pronounced for stool parameters. Stool frequency is an objective parameter, and visual scoring of consistency is to some extent more objective, particularly when true changes occur (e.g. from hard to normal stools). Therefore, an increase in stool frequency was observed for the EpiCor-treated groups, and this effect was nearly significant in the total cohort, whereas stool consistency improved significantly in the total population and in the severe subgroup, within the first 2 weeks of treatment (Fig. 4).

The impact of constipation on quality of life is pertinent and comparable to that caused by serious chronic conditions such as osteoarthritis and diabetes [54]. Therefore, quality of life parameters are valid assessments to consider in these type of studies [10, 13]. PAC-QOL, which focuses on the effects of constipation on quality of life, showed better results for EpiCor fermentate. Although not reaching significance, ‘between’ groups’ analysis has shown that all domains of this instrument improve in the EpiCor-treated moderate subgroup when compared to placebo, and a nearly significant effect was detected for physical discomfort (Fig. 5a). This suggests that an improvement of GI comfort and bowel movements has a direct impact on patient’s quality of life. The improvement reported for physical discomfort may be related to the improvement observed for bloating/distension, feeling of fullness and stool frequency, as an increase in bowel movements may result in a less bloated/full feeling, which in turn can lead to the perception that physical discomfort improves. In theory, disease-specific quality of life questionnaires, which evaluate problems specific to the FGIDs in question, can detect smaller and more relevant changes in health status, which are otherwise missed by generic instruments [10]. Nevertheless, the generic perceived stress instrument used in this study has also shown a positive improvement in general stress scores within the total cohort and moderate subgroup, particularly at the end of the study (Fig. 5b).

### Changes in gut microbial composition

Gastrointestinal motility and gut microbiota are clearly associated [23]. Experiments performed in humanized germ-free mice suggest that, on the one hand, gut microbes modulate bowel movements and, on the other hand, changes in GI motility modify the resident microbial population [24]. The ecological principles of r/K selection have been proposed to explain this: as GI transit time decreases (e.g. during diarrhea) species that are better adapted to grow rapidly during reduced competition (r-selected) will dominate. In contrast, as GI transit time increases (as during constipation) the community will be dominated by species that grow more slowly in unrestricted conditions but that are better adapted to persist in a competitive environment (K-selected) [24]. Because the microbiome is metabolically interconnected, direct effects of motility on key groups of bacteria may result in a cascade of events with broader consequences to the equilibrium of this ecosystem. Upstream of this interplay between gut microbiome and GI transit is diet, and dietary habits influence GI transit in a microbiota dependent- and independent-manner [24]. For example, the bulk effect attributed to fibers is well recognized, and is behind the reason why an increase in fiber intake is recommended by practitioners to ameliorate constipation. Obviously, fiber consumption and intake of over-the-counter laxatives or antidiarrheals will influence GI transit time. In turn, as mentioned above, GI transit time has a selective role on microbiome composition. Interestingly, both cellulose (poorly fermented by the gut microbiota) and polyethylene glycol (PEG), the most widely used compound in a number of commercial laxatives, were shown to accelerate GI transit time in humanized-mice with a concomitant increase in Bacteroidales and Bacteroidaceae [24]. In contrast, treatment with loperamide (Imodium®, antidiarrheal) was shown to delay transit time with the consequent increase in Porphyromonadaceae [24]. These studies suggest that these families of bacteria have different adaptation mechanisms that distinctively influence their success relatively to gut transit [24]. In addition, fibers or other dietary supplements with prebiotic-like characteristics modulate the gut microbiome and, depending on the type of substrate, will offer nutritional advantage to certain groups of bacteria that are able to degrade it. In consequence, this will lead to the production of metabolites, such as SCFA, that are known to regulate GI transit [25, 55]. Moreover, the gut microbiota is also able to directly modulate endocrine cells in the gastrointestinal mucosa to produce molecules that influence gut motor function, such as gastrin, serotonin (*in* [24]) and the satiety hormones Peptide YY (PYY) and Glucagon-like peptide (GLP)-1 (reviewed in [56, 57]).

Despite the recognized role of the gut microbiome in a disorder as common as constipation, very little is known about either quantitative or qualitative changes of bacteria in this condition [53]. Several reasons may account for this [28, 53]: 1) lack of in-depth studies using for example sequencing technology. Some studies have been done by using either quantitative (q)PCR or conventional culture-based techniques, which offer very limited insight to the extended differences between constipated and healthy individuals; 2) the use of different techniques may influence outcome, and so direct comparison between studies is not straightforward; 3) microbial analysis is typically performed in fecal samples, however, the community present in feces may substantially differ from mucosa-associated microbiota; 4) constipation is a heterogeneous condition, subject to large interindividual symptomatology. The existence of subgroups of patients exhibiting different microbial signatures has been hypothesized; 5) gut microbiome composition is largely individual-dependent and so, if only minor changes in particular species are to be expected in constipation, these may be masked by a high interindividual variability; 6) gut microbiome composition and constipation are largely affected by diet, and so regional differences are also to be expected, which may hamper direct comparison between studies; 7) studies have been done in a limited number of individuals, which under-powers statistical tests, and 8) a general lack of attention has been given to the role of the gut microbiome in constipation. Most studies have been performed in IBS patients, which may or not suffer from constipation. However, and despite some controversy [58], they are different diseases in etiology and extrapolation has to be done cautiously.

Nevertheless, constipation is a common condition in infants and so has deserved some attention in the past few years. In a study using conventional culturing techniques, Zoppi and colleagues [40] have reported higher numbers of *Clostridium* and *Bifidobacterium* species among children with constipation. Similarly to these findings, in another study using a PCR-based profiling method, a higher relative abundance of bifidobacteria, particularly *Bifidobacterium longum*, has been observed in constipated children [28]. However, a recent study using 16 s RNA pyrosequencing has found a lower abundance of Bacteroidetes (mostly *Prevotella* species) in constipated obese children, whereas several families and genera belonging to the phylum Firmicutes were higher [31]. In adults, reports are even scarcer. In a study performed in 57 adult patients suffering from constipation and using conventional culturing techniques, abundance of *Bifidobacterium* and *Lactobacillus* was found to be significantly lower in constipated subjects [30]. Similarly to these findings, in a study examining the gut microbiome of constipation-predominant IBS patients (C-IBS), both *Bifidobacterium* and *Lactobacillus* numbers were found to be lower in C-IBS when compared to healthy subjects. In addition, the numbers of butyrate-producing *Roseburia/Eubacterium rectale* group were also significantly lower in these patients [27]. However, in a recent study investigating the effect of a probiotic treatment in patients with functional constipation, *Bifidobacterium* and *Bacteroides* species were found to be significantly less abundant in constipated patients, whereas the proportion of *Lactobacillus*, *Escherichia coli* and *Clostridium* remained unchanged [59]. It is clear that there is a discrepancy between studies, and most of them have been done using limited techniques. Therefore, an in-depth study showing the differences between gut microbiome composition of healthy and constipated individuals is still lacking.

In the present study we have found that EpiCor treatment increases the relative numbers of Bacteroidetes (allied to a decrease in F/B ratio), particularly in the severe subgroup (Fig. 6), and this seems to be due to an increase in members of Bacteroidaceae, Porphyromonadaceae and Prevotellaceae, namely *Bacteroides, Barnesiella&Odoribacter* and *Prevotella*, respectively (Additional file: 7). Interestingly, a lower incidence of *Prevotella* species has been hypothesized to be associated with a low-fiber diet and insufficient plant-based foods consumption [60, 61], and so to be a major cause of dysbiosis in the gut of constipated patients [31]. Therefore, an increase in their numbers by means of probiotic-like supplements has been proposed for the management of constipation [31]. A similar result has been reported in C-IBS patients. Rajilić-Stojanović and colleagues (2011) have found that C-IBS patients have a 2-fold increased ratio of Firmicutes to Bacteroidetes when compared to healthy individuals. This deficit in Bacteroidetes numbers was related to a lower incidence of members of the order Bacteroidales, including *Allistipes*, *Bacteroides*, *Odoribacter*, *Parabacteroides* and *Prevotella* [62]. In addition, antidiarrheal treatment of humanized germ-free mice also led to an increase in the Firmicutes to Bacteroidetes ratio, and treatment with PEG (laxative) and cellulose increased the relative numbers of Bacteroidaceae [24]. Altogether, these and our results suggest that delayed GI transit is associated with decreased Bacteroidetes numbers (and concomitant increase in F/B ratio) whereas acceleration of GI transit increases the relative numbers of Bacteroidetes, namely of members of the Bacteroidaceae and Prevotellaceae groups. Therefore, the results here obtained at the level of the gut microbiome, i.e., a significant increase in *Bacteroides* and *Prevotella* species may explain the positive effects on stool frequency and consistency observed for the EpiCor-treated groups, particularly for the severe subgroup (Fig. 4). Nevertheless, and in contrast with the results obtained using humanized germ-free mice [24], here we have found an increase in Porphyromonadaceae upon EpiCor intake, thus stressing the complexity of the ecological equilibrium of the gut microbiome and the difficulties in comparing studies using different models and techniques. Also interestingly, some of these changes were less pronounced or absent in the moderate subgroup, while others seem to be specific for this cohort (Additional file: 8). For instance, a significant increase in *Akkermansia muciniphila* was observed within the moderate subgroup. This important mucin degrader, that resides in the intestinal mucus layer, has been shown to be important for proper gut functioning and to inversely correlate with metabolic disorders [63]. In addition, we have found that the relative abundance of *Blautia* and *Roseburia* significantly decreased in the moderate subgroup, and these two groups of bacteria have also been described to be higher in IBS and C-IBS patients [62]. Moreover, *Blautia* numbers are positively and strongly associated with IBS symptoms scores and so a decrease in their numbers may be considered beneficial in the context of IBS [62]. However, whether these changes correlate with improved GI symptoms such as bloating, which were reported by the moderate subgroup (Fig. 3), is not known and demands further research. In contrast, some groups are only increasing within the severe subgroup, such as *Anaerostipes*, a genus containing acetate- and lactate-consuming, butyrate-producing bacteria, with recognized health-enhancing effects [64] (Additional file: 7). Once more these results support subgroup analysis and suggest that patients experiencing different gradations of GI discomfort may actually have also different levels of dysbiosis, and this ultimately may advocate for a differentiated treatment.

Management of constipation can be complex and may require multimodal therapy that follows a step-down approach [54]. One of the first recommendations by physicians is to accelerate colonic transit by adequate fiber intake or use of bulk-forming agents. These will retain water in the stools allowing them to pass more easily. When such approaches are ineffective, the use of hyperosmotic agents such as glycerin or sorbitol and stimulants such as senna or bisacodyl must be considered [54], but these have undesirable side effects and mustn’t be used for prolonged periods of time. Therefore, some new and less harmful approaches have been suggested [54], such as administration of probiotics [65], but due to a lack of properly controlled trials recommendation is still poor [54].

It is not totally clear what may be the mechanism behind EpiCor’s beneficial effect on constipation, but a prebiotic-like effect cannot be discarded. The findings that the gut microbiome of constipated patients is dysbiotic and the reported in vitro positive effects of EpiCor on gut luminal environment and microbial composition [21] suggest that EpiCor could have a favorable effect on constipation through modulation of the gut microbial community, by increasing the numbers of beneficial bacterial groups. This same in vitro study has also shown that EpiCor fermentation results in an increase in butyrate levels [21], and butyrate has been described as essential for optimal ileal and colonic motor activity [23, 25, 26]. Therefore, the positive effects of EpiCor on constipation could be mediated by an increase in butyrate production, but this demands further research. Hence, it is possible that the changes in microbial composition observed in this study are, as previously reported, secondary to constipation [30]. In this manner, EpiCor intake, by increasing bowel movements (by for example increasing butyrate levels or other metabolites), would result in changes in the composition of the gut microbial community in an indirect manner.

## Conclusions

Despite its low daily dose (500 mg/day), this study suggests that EpiCor fermentate has a positive effect on GI symptoms and stool parameters in individuals with symptoms of gastrointestinal discomfort and reduced bowel movements. Improvement of these symptoms was nicely correlated with improved quality of life and reduced stress levels. Moreover, EpiCor fermentate led to changes in gut microbial composition that parallel observations done by others in constipated and C-IBS patients. Naturally, conclusions based on a single study and with subgroup analysis are to be treated with caution, and it is formally recommended to obtain final proof in a second confirmatory trial in order to substantiate a health claim on ‘gastrointestinal discomfort’.

## Additional files

Additional file 1: Mean values and standard error or the mean (SEM) calculated for the daily records on GI symptoms (averages were calculated for 2-week intervals). (PDF 459 kb)

Additional file 2: Mean values and standard error or the mean (SEM) calculated for the daily records of stool frequency and consistency (averages were calculated for 2-week intervals). (PDF 417 kb)

Additional file 3: Mean values and standard error or the mean (SEM) calculated for the questionnaires PAC-QOL and PSS at visits 1, 2 and 3. (PDF 453 kb)

Additional file 4: Pareto-Lorenz curve representing the cumulative number of species relatively to their cumulative abundance. If all species would be equally distributed they would follow the straight line of equal distribution. However, this is not the case. Only a limited number of species is over represented with in the microbiome accounting for nearly 100% of relative abundance. Due to the fact that the remaining OTUs are so low abundant, we can assume that they have little expression interms of physiological outcome, and so they can be discarded from the analysis. (PDF 298 kb)

Additional file 5: Phyla relative abundances (%) within the total cohort (a) and the two subgroups, severe (b) and moderate (c) that have been treated either with placebo or EpiCor . V1, V2 and V3 correspond to visit 1 (baseline), visit 2 (3-weeks after treatment) and visit 3 (6-weeks after treatment), respectively. (PDF 54 kb)

Additional file 6: Microbial taxa fold-changes (V2/V1 and V3/V1) in the total cohort. (PDF 554 kb)

Additional file 7: Microbial taxa fold-changes (V2/V1 and V3/V1) in the severe subgroup. (PDF 554 kb)

Additional file 8: Microbial taxa fold-changes (V2/V1 and V3/V1) in the moderate subgroup. (PDF 551 kb)

## Abbreviations

ANOVA: Analysis of variance
CI: Confidence interval
C-IBS: Constipation-predominant IBS
CIC: Chronic idiopathic constipation
DTS: Daily total score
EFSA: European food safety authority
F/B: Firmicutes/Bacteroidetes ratio
FC: Fold-change
FGIDs: Functional gastrointestinal disorders
GI: Gastrointestinal
HCL: Hierarchical clustering
IBS: Irritable bowel syndrome
ITS: Instrument total score
OTU: Operational taxonomic unit
PAC-QOL: Patient assessment of constipation quality of life
PCA: Principal component analysis
PEG: Polyethylene glycol
PSS: Perceived stress scale
SCFA: Short-chain fatty acids
V: Visit
