# Supplementary material for: A yeast fermentate improves gastrointestinal discomfort and constipation by modulation of the gut microbiome: results from a randomized double-blind placebo-controlled pilot trial
Source: BMC Complement Altern Med. 2017 Sep 4;17:441. doi: 10.1186/s12906-017-1948-0 (PMC5584023; doi:10.1186/s12906-017-1948-0)
Supplement: Supplementary file 1 — Mean values and standard error or the mean (SEM) calculated for the daily records on GI symptoms (averages were calculated for 2-week intervals). (PDF 459 kb) [file 12906_2017_1948_MOESM1_ESM.pdf]

Additional file 2 Mean values and standard error or the mean (SEM) calculated for the daily records on GI symptoms (averages were calculated for 2-week intervals).

GI symptoms		Total cohort		Severe		Moderate	
		EpiCor	Placebo	EpiCor	Placebo	EpiCor	Placebo
**BD_T1**	**Mean**	1.72	1.51	2.05	1.91	0.95	0.69
	**SEM**	±0.14	±0.13	±0.16	±0.13	±0.14	±0.09
**BD_T2**	**Mean**	1.30	1.24	1.60	1.46	0.61	0.79
	**SEM**	±0.14	±0.14	±0.17	±0.18	±0.11	±0.15
**BD_T3**	**Mean**	1.24	1.23	1.55	1.47	0.51	0.73
	**SEM**	±0.15	±0.14	±0.19	±0.18	±0.11	±0.15
**BD_T4**	**Mean**	1.21	1.18	1.51	1.42	0.51	0.68
	**SEM**	±0.16	±0.14	±0.19	±0.17	±0.13	±0.21
**BD**	***p*-value (ANOVA)**	***<0.0001***	***0.003***	***0.002***	***<0.0001***	***0.001***	*0.873*
**GAS_T1**	**Mean**	1.82	1.65	2.11	1.90	1.14	1.12
	**SEM**	±0.14	±0.14	±0.17	±0.17	±0.18	±0.20
**GAS_T2**	**Mean**	1.66	1.36	1.98	1.54	0.92	0.97
	**SEM**	±0.15	±0.14	±0.18	±0.17	±0.13	±0.19
**GAS_T3**	**Mean**	1.64	1.36	1.91	1.51	1.02	1.04
	**SEM**	±0.16	±0.14	±0.20	±0.17	±0.18	±0.21
**GAS_T4**	**Mean**	1.56	1.29	1.76	1.50	1.07	0.85
	**SEM**	±0.17	±0.13	±0.21	±0.16	±0.21	±0.19
**GAS**	***p*-value (ANOVA)**	*0.099*	***<0.0001***	*0.078*	***0.000***	*0.485*	*0.198*
**RUM_T1**	**Mean**	1.33	1.34	1.63	1.63	0.65	0.73
	**SEM**	±0.13	±0.13	±0.16	±0.14	±0.11	±0.16
**RUM_T2**	**Mean**	1.14	1.10	1.45	1.31	0.41	0.65
	**SEM**	±0.13	±0.12	±0.15	±0.15	±0.13	±0.11
**RUM_T3**	**Mean**	1.07	1.08	1.35	1.26	0.43	0.70
	**SEM**	±0.15	±0.11	±0.18	±0.15	±0.13	±0.11
**RUM_T4**	**Mean**	0.95	1.04	1.21	1.29	0.36	0.51
	**SEM**	±0.14	±0.12	±0.16	±0.15	±0.16	±0.12
**RUM**	***p*-value (ANOVA)**	***0.002***	***0.005***	***0.015***	***0.011***	*0.114*	*0.165*
**FF_T1**	**Mean**	1.54	1.34	1.88	1.73	0.76	0.53
	**SEM**	±0.15	±0.13	±0.17	±0.13	±0.16	±0.10
**FF_T2**	**Mean**	1.10	1.05	1.41	1.22	0.38	0.69
	**SEM**	±0.15	±0.13	±0.18	±0.18	±0.10	±0.14
**FF_T3**	**Mean**	1.09	1.04	1.35	1.21	0.48	0.67
	**SEM**	±0.16	±0.13	±0.20	±0.17	±0.13	±0.15
**FF_T4**	**Mean**	1.03	0.97	1.30	1.15	0.40	0.60
	**SEM**	±0.16	±0.13	±0.21	±0.17	±0.13	±0.18
**FF**	***p*-value (ANOVA)**	***<0.0001***	***0.004***	***0.002***	***0.000***	***0.004***	*0.618*
**AD_T1**	**Mean**	0.81	0.73	1.05	0.91	0.27	0.34
	**SEM**	±0.14	±0.11	±0.18	±0.14	±0.09	±0.08
**AD_T2**	**Mean**	0.64	0.49	0.81	0.61	0.24	0.25
	**SEM**	±0.12	±0.10	±0.16	±0.13	±0.06	±0.12
**AD_T3**	**Mean**	0.59	0.48	0.77	0.62	0.16	0.20
	**SEM**	±0.13	±0.08	±0.17	±0.11	±0.06	±0.05

**AD_T4**	**Mean**	0.59	0.48	0.76	0.64	0.18	0.15
	**SEM**	±0.13	±0.08	±0.17	±0.10	±0.06	±0.05
**AD**	***p*-value (ANOVA)**	***0.021***	***0.011***	***0.032***	***0.035***	*0.508*	*0.213*
**DTS_T1**	**Mean**	7.20	6.56	8.68	8.08	3.74	3.41
	**SEM**	±0.56	±0.46	±0.60	±0.42	±0.31	±0.32
**DTS_T2**	**Mean**	5.84	5.23	7.24	6.13	2.56	3.35
	**SEM**	±0.59	±0.47	±0.69	±0.57	±0.28	±0.52
**DTS_T3**	**Mean**	5.63	5.18	6.93	6.07	2.59	3.33
	**SEM**	±0.64	±0.45	±0.78	±0.57	±0.39	±0.44
**DTS_T4**	**Mean**	5.33	4.96	6.53	6.00	2.53	2.79
	**SEM**	±0.65	±0.47	±0.80	±0.54	±0.49	±0.55
**DTS**	***p*-value (ANOVA)**	***<0.0001***	***<0.0001***	***0.001***	***<0.0001***	***0.027***	*0.381*

Legend: The enlisted gastrointestinal (GI) symptoms are BD (bloating/distension), GAS (passage of gas), RUM (GI rumbling), FF (feeling of fullness) and AD (abdominal discomfort). A daily total score (DTS) calculated as the sum of all items recorded each day is also shown. The periods depicted are T1 (average of weeks 1 and 2 of run-in), T2 (average of weeks 1 and 2 of intervention), T3 (average of weeks 3 and 4 of intervention) and T4 (average of weeks 5 and 6 of intervention). A lower score is concomitant with lower severity of symptoms. A one-way repeated measures ANOVA was used to test for significant changes over time within each group (p-values ≤ 0.05 are depicted in bold text).
