# Supplementary material for: A yeast fermentate improves gastrointestinal discomfort and constipation by modulation of the gut microbiome: results from a randomized double-blind placebo-controlled pilot trial
Source: BMC Complement Altern Med. 2017 Sep 4;17:441. doi: 10.1186/s12906-017-1948-0 (PMC5584023; doi:10.1186/s12906-017-1948-0)
Supplement: Supplementary file 2 — Mean values and standard error or the mean (SEM) calculated for the daily records of stool frequency and consistency (averages were calculated for 2-week intervals). (PDF 417 kb) [file 12906_2017_1948_MOESM2_ESM.pdf]

Additional file 3 Mean values and standard error or the mean (SEM) calculated for the daily records of stool frequency and consistency (averages were calculated for 2-week intervals).

GI symptoms		Total cohort		Severe		Moderate	
		EpiCor	Placebo	EpiCor	Placebo	EpiCor	Placebo
SF_T1	Mean	0.68	0.66	0.69	0.60	0.67	0.79
	SEM	±0.03	±0.05	±0.05	±0.06	±0.05	±0.06
SF_T2	Mean	0.88	0.76	0.87	0.70	0.90	0.89
	SEM	±0.05	±0.06	±0.06	±0.09	±0.11	±0.07
SF_T3	Mean	0.89	0.78	0.91	0.72	0.87	0.90
	SEM	±0.05	±0.05	±0.07	±0.06	±0.07	±0.07
SF_T4	Mean	0.86	0.79	0.90	0.72	0.79	0.92
	SEM	±0.05	±0.05	±0.07	±0.06	±0.09	±0.05
SF	*p*-value (ANOVA)	***<0.0001***	***0.003***	***0.000***	***0.040***	***0.005***	*0.113*
SC_T1	Mean	2.88	3.29	2.68	3.18	3.34	3.53
	SEM	±0.17	±0.16	±0.21	±0.20	±0.25	±0.28
SC_T2	Mean	3.22	3.18	3.08	3.07	3.52	3.40
	SEM	±0.17	±0.17	±0.21	±0.20	±0.26	±0.30
SC_T3	Mean	3.03	3.25	2.99	3.29	3.11	3.18
	SEM	±0.17	±0.16	±0.21	±0.21	±0.31	±0.21
SC_T4	Mean	2.95	3.29	2.87	3.31	3.13	3.25
	SEM	±0.15	±0.15	±0.18	±0.19	±0.30	±0.25
SC	*p*-value (ANOVA)	***0.037***	*0.535*	***0.044***	*0.424*	***0.031***	*0.425*

Legend: SC, stool consistency; SF, stool frequency. The periods depicted are T1 (average of weeks 1 and 2 of run-in), T2 (average of weeks 1 and 2 of intervention), T3 (average of weeks 3 and 4 of intervention) and T4 (average of weeks 5 and 6 of intervention). A higher score is concomitant with an increase in frequency and normalization of consistency. A one-way repeated measures ANOVA was used to test for significant changes over time within each group (p-values ≤ 0.05 are depicted in bold text).
