# Supplementary material for: A yeast fermentate improves gastrointestinal discomfort and constipation by modulation of the gut microbiome: results from a randomized double-blind placebo-controlled pilot trial
Source: BMC Complement Altern Med. 2017 Sep 4;17:441. doi: 10.1186/s12906-017-1948-0 (PMC5584023; doi:10.1186/s12906-017-1948-0)
Supplement: Supplementary file 3 — Mean values and standard error or the mean (SEM) calculated for the questionnaires PAC-QOL and PSS at visits 1, 2 and 3. (PDF 453 kb) [file 12906_2017_1948_MOESM3_ESM.pdf]

Additional file 4 Mean values and standard error or the mean (SEM) calculated for the questionnaires PAC-QOL and PSS at visits 1, 2 and 3.

Instrument	GI symptoms		Total cohort		Severe		Moderate	
			**EpiCor**	**Placebo**	**EpiCor**	**Placebo**	**EpiCor**	**Placebo**
**PAC-QOL**	**PhD_V1**	**Mean**	1.51	1.39	1.69	1.65	1.10	0.87
		**SEM**	±0.12	±0.10	±0.14	±0.10	±0.17	±0.13
	**PhD_V2**	**Mean**	1.05	1.17	1.22	1.33	0.65	0.83
		**SEM**	±0.12	±0.12	±0.15	±0.14	±0.14	±0.19
	**PhD_V3**	**Mean**	1.04	1.12	1.21	1.32	0.67	0.69
		**SEM**	±0.12	±0.11	±0.15	±0.14	±0.16	±0.16
	**PhD**	***p*-value (ANOVA)**	***<0.0001***	***0.005***	***0.001***	***0.007***	***0.017***	*0.435*
	**PsD_V1**	**Mean**	0.68	0.65	0.78	0.78	0.45	0.38
		**SEM**	±0.08	±0.08	±0.11	±0.09	±0.11	±0.11
	**PsD_V2**	**Mean**	0.45	0.45	0.50	0.50	0.33	0.35
		**SEM**	±0.07	±0.06	±0.09	±0.08	±0.09	±0.07
	**PsD_V3**	**Mean**	0.38	0.39	0.46	0.48	0.17	0.19
		**SEM**	±0.07	±0.07	±0.09	±0.09	±0.06	±0.07
	**PsD**	***p*-value (ANOVA)**	***<0.0001***	***0.001***	***0.001***	***0.002***	***0.027***	*0.129*
	**W&C_V1**	**Mean**	1.05	1.03	1.17	1.22	0.78	0.64
		**SEM**	±0.09	±0.08	±0.11	±0.09	±0.16	±0.14
	**W&C_V2**	**Mean**	0.79	0.77	0.83	0.87	0.67	0.57
		**SEM**	±0.08	±0.09	±0.09	±0.11	±0.17	±0.13
	**W&C_V3**	**Mean**	0.69	0.63	0.81	0.72	0.43	0.45
		**SEM**	±0.08	±0.08	±0.10	±0.09	±0.09	±0.13
	**W&C**	***p*-value (ANOVA)**	***0.000***	***<0.0001***	***0.002***	***<0.0001***	*0.090*	*0.112*
	**SAT_V1**	**Mean**	2.62	2.50	2.69	2.65	2.45	2.19
		**SEM**	±0.13	±0.10	±0.15	±0.12	±0.25	±0.16
	**SAT_V2**	**Mean**	2.13	2.28	2.26	2.41	1.84	2.03
		**SEM**	±0.16	±0.14	±0.19	±0.17	±0.27	±0.23
	**SAT_V3**	**Mean**	1.99	2.04	2.13	2.18	1.64	1.75
		**SEM**	±0.14	±0.14	±0.16	±0.15	±0.22	±0.27
	**SAT**	***p*-value (ANOVA)**	***0.000***	***0.004***	***0.010***	***0.027***	***0.013***	*0.166*
	**ITS_V1**	**Mean**	1.27	1.23	1.39	1.40	1.01	0.86
		**SEM**	±0.08	±0.07	±0.10	±0.07	±0.14	±0.10
	**ITS_V2**	**Mean**	0.96	1.00	1.04	1.10	0.77	0.79
		**SEM**	±0.08	±0.07	±0.09	±0.09	±0.13	±0.12
	**ITS_V3**	**Mean**	0.88	0.88	1.00	1.00	0.60	0.64
		**SEM**	±0.08	±0.07	±0.09	±0.08	±0.10	±0.11
	**ITS**	***p*-value (ANOVA)**	***<0.0001***	***<0.0001***	***<0.0001***	***<0.0001***	***0.022***	***0.040***
**PSS**	**ITS_V1**	**Mean**	11.92	11.98	11.96	13.44	11.83	8.92
		**SEM**	±1.05	±0.99	±1.26	±1.26	±1.96	±1.26
	**ITS_V2**	**Mean**	11.68	11.58	11.36	12.67	12.42	9.31
		**SEM**	±0.99	±1.07	±1.26	±1.29	±1.56	±1.84
	**ITS_V3**	**Mean**	10.13	11.95	10.43	12.41	9.42	11.00
		**SEM**	±1.19	±1.00	±1.46	±1.07	±2.12	±2.18
	**ITS**	***p*-value (ANOVA)**	***0.016***	*0.846*	***0.044***	*0.555*	*0.197*	*0.379*

Legend: The enlisted Patient Assessment of Constipation Quality of Life (PAC-QOL) items are PhD (physical discomfort), PsD (psychosocial discomfort), W&C (worries and concerns) and SAT (satisfaction). An instrument total score (ITS) calculated as the average of all 28 items recorded at each visit is also shown. The Perceived stress scale

(PSS) instrument only includes a total score (ITS) calculated as the sum of all 10 items recorded at each visit. Both instruments were filled in during visits 1 (V1, baseline), V2 and V3. A lower score is concomitant with a better quality of life and lower perceived stress for PAC-QOL and PSS, respectively. A one-way repeated measures ANOVA was used to test for significant changes over time within each group (p-values ≤ 0.05 are depicted in bold text).
