# Supplementary material for: A yeast fermentate improves gastrointestinal discomfort and constipation by modulation of the gut microbiome: results from a randomized double-blind placebo-controlled pilot trial
Source: BMC Complement Altern Med. 2017 Sep 4;17:441. doi: 10.1186/s12906-017-1948-0 (PMC5584023; doi:10.1186/s12906-017-1948-0)
Supplement: Supplementary file 4 — Pareto-Lorenz curve representing the cumulative number of species relatively to their cumulative abundance. If all species would be equally distributed they would follow the straight line of equal distribution. However, this is not the case. Only a limited number of species is over represented with in the microbiome accounting for nearly 100% of relative abundance. Due to the fact that the remaining OTUs are so low abundant, we can assume that they have little expression interms of physiological outcome, and so they can be discarded from the analysis. (PDF 298 kb) [file 12906_2017_1948_MOESM4_ESM.pdf]

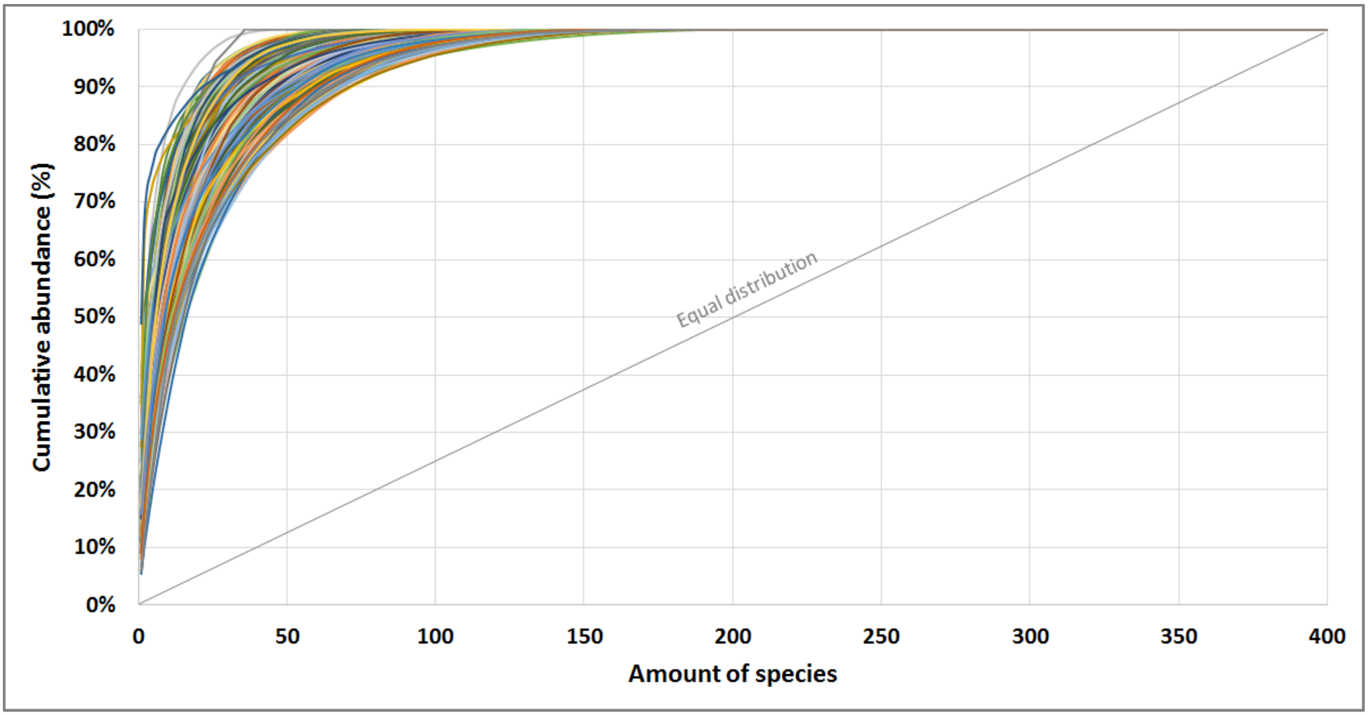

Additional file 5 Pareto-Lorenz curve representing the cumulative number of species relatively to their cumulative abundance. If all species would be equally distributed they would follow the straight line of equal distribution. However, this is not the case. Only a limited number of species is overrepresented within the microbiome accounting for nearly 100% of relative abundance. Due to the fact that the remaining OTUs are so low abundant, we can assume that they have little expression in terms of physiological outcome, and so they can be discarded from the analysis.
