# Supplementary material for: A yeast fermentate improves gastrointestinal discomfort and constipation by modulation of the gut microbiome: results from a randomized double-blind placebo-controlled pilot trial
Source: BMC Complement Altern Med. 2017 Sep 4;17:441. doi: 10.1186/s12906-017-1948-0 (PMC5584023; doi:10.1186/s12906-017-1948-0)
Supplement: Supplementary file 5 — Phyla relative abundances (%) within the total cohort (a) and the two subgroups, severe (b) and moderate (c) that have been treated either with placebo or EpiCor . V1, V2 and V3 correspond to visit 1 (baseline), visit 2 (3-weeks after treatment) and visit 3 (6-weeks after treatment), respectively. (PDF 54 kb) [file 12906_2017_1948_MOESM5_ESM.pdf]

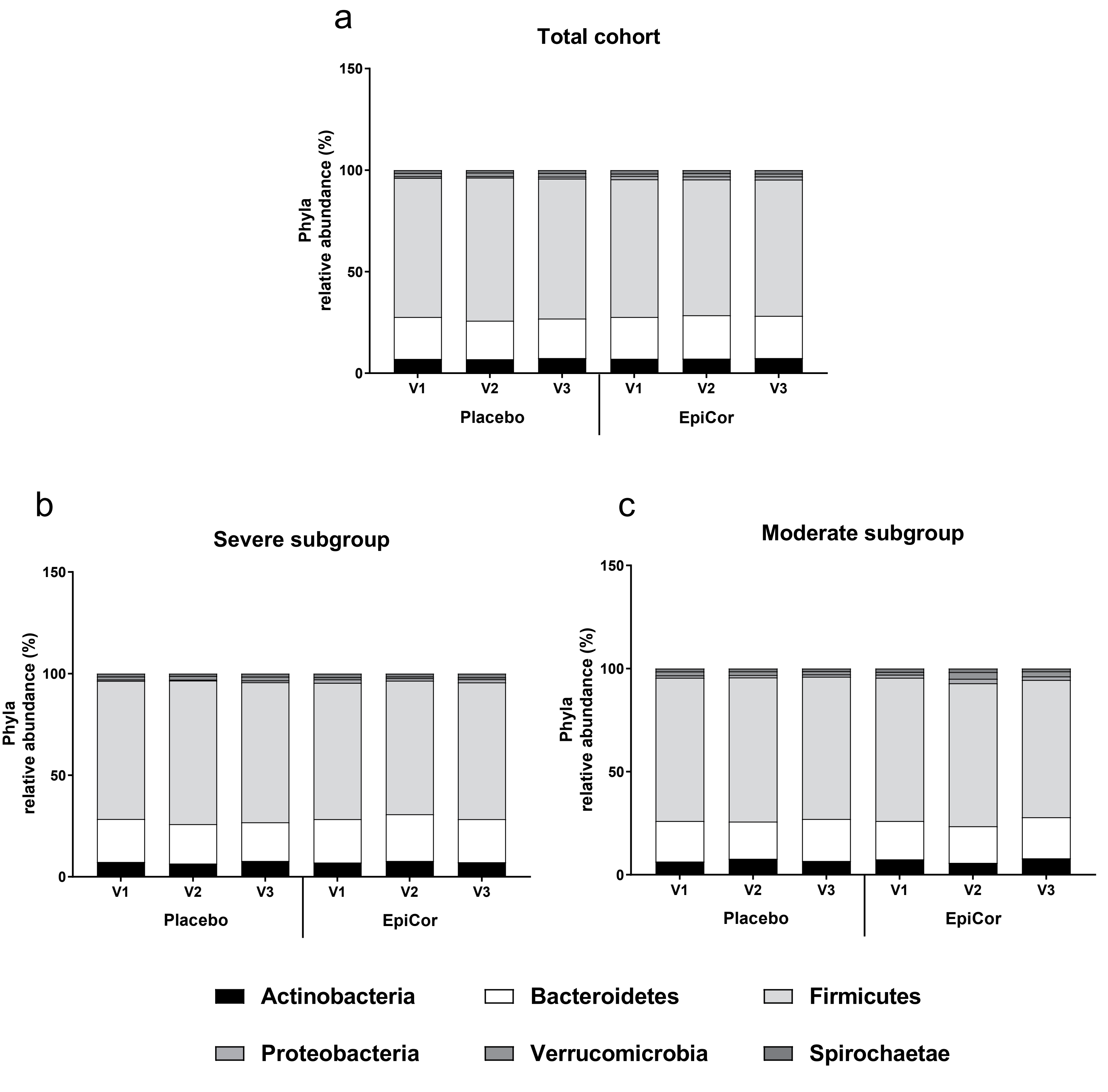

Additional file 6 Phyla relative abundances (%) within the total cohort (a) and the two subgroups, severe (b) and moderate (c) that have been treated either with placebo or EpiCor . V1, V2 and V3 correspond to visit 1 (baseline), visit 2 (3-weeks after treatment) and visit 3 (6-weeks after treatment), respectively.
