# Supplementary material for: A yeast fermentate improves gastrointestinal discomfort and constipation by modulation of the gut microbiome: results from a randomized double-blind placebo-controlled pilot trial
Source: BMC Complement Altern Med. 2017 Sep 4;17:441. doi: 10.1186/s12906-017-1948-0 (PMC5584023; doi:10.1186/s12906-017-1948-0)
Supplement: Supplementary file 6 — Microbial taxa fold-changes (V2/V1 and V3/V1) in the total cohort. (PDF 554 kb) [file 12906_2017_1948_MOESM6_ESM.pdf]

Additional file 7 Microbial taxa fold-changes (V2/V1 and V3/V1) in the total cohort.

Taxa		Total cohort (n=79)							
		Placebo (n=40)				EpiCor (n=39)			
		V2/V1		V3/V1		V2/V1		V3/V1	
		FC	*p*-val.	FC	*p*-val.	FC	*p*-val.	FC	*p*-val.
**Phylum Actinobacteria**									
**Bifidobacteriaceae**	-	0.88	*0.536*	0.97	*0.952*	0.95	*0.885*	1.02	*0.979*
	Bifidobacterium	0.88	*0.296*	0.97	*0.906*	0.95	*0.776*	1.02	*0.957*
**Coriobacteriaceae**	-	1.13	*0.753*	1.20	*0.507*	1.07	*0.908*	1.07	*0.896*
	Asaccharobacter	1.35	*0.990*	2.46	*0.842*	1.40	*0.928*	1.67	*0.815*
	Collinsella	1.35	*0.306*	1.34	*0.330*	0.80	*0.699*	0.65	*0.352*
	Coriobacteriaceae_uncl.	0.70	*0.771*	0.99	*0.999*	1.53	*0.456*	1.98	***0.085***
	Eggerthella	2.60	*1.000*	1.87	*1.000*	0.53	*0.997*	0.52	*0.997*
	Enterorhabdus	1.34	*0.985*	0.52	*0.972*	0.50	*0.949*	0.63	*0.972*
	Slackia	1.09	*0.999*	0.55	*0.983*	1.23	*0.974*	0.32	*0.805*
**Propionibacteriaceae**	-	0.05	*0.994*	0.01	*1.000*	2.65	*0.993*	1.48	*0.999*
	Propionibacterium	0.05	*0.988*	0.01	*1.000*	2.65	*0.985*	1.48	*0.999*
**Phylum Bacteroidetes**									
**Bacteroidaceae**	-	0.99	*0.973*	1.07	*0.463*	1.06	*0.480*	1.08	*0.293*
	Bacteroides	0.99	*0.946*	1.07	*0.224*	1.06	*0.231*	1.08	***0.088***
**Bacteroidetes_uncl.**	-	1.29	*0.944*	1.07	*0.996*	0.99	*1.000*	0.76	*0.888*
**Bacteroidales_uncl.**	-	1.43	*0.968*	0.86	*0.997*	1.20	*0.997*	1.94	*0.936*
**Porphyromonadaceae**	-	0.71	***0.004***	0.79	***0.050***	1.09	*0.666*	0.99	*0.998*
	Barnesiella	0.64	*0.199*	0.65	*0.227*	1.39	*0.201*	1.36	*0.262*
	Butyricimonas	1.56	*0.987*	1.54	*0.988*	0.72	*0.950*	0.88	*0.991*
	Odoribacter	0.72	*0.977*	1.16	*0.993*	1.30	*0.989*	1.26	*0.992*
	Porphyromonadaceae_uncl.	0.90	*0.852*	0.97	*0.983*	1.08	*0.907*	0.89	*0.846*
	Parabacteroides	0.62	***0.099***	0.67	*0.170*	0.78	*0.718*	0.67	*0.491*
**Prevotellaceae**	-	0.80	*0.464*	0.98	*0.988*	1.33	*0.186*	1.11	*0.796*
	Alloprevotella	1.07	*0.999*	1.44	*0.951*	0.74	*0.994*	0.81	*0.997*
	Hallella	1.00	*1.000*	0.00	*1.000*	0.00	*0.937*	0.00	*0.937*
	Paraprevotella	0.02	*0.723*	0.01	*0.718*	1.71	*0.729*	0.14	*0.633*
	Prevotella	0.82	*0.495*	1.04	*0.955*	1.48	***0.075***	1.43	*0.121*
	Prevotellaceae_uncl.	1.16	*0.986*	0.99	*1.000*	1.02	*0.999*	1.12	*0.982*
**Rikenellaceae**	-	1.10	*0.721*	0.86	*0.563*	0.70	***0.093***	0.80	*0.320*
	Alistipes	1.10	*0.523*	0.86	*0.326*	0.70	***0.009***	0.80	*0.105*
**Phylum Firmicutes**									
**Acidaminococcaceae**	-	1.40	*0.177*	1.63	***0.017***	0.93	*0.911*	1.04	*0.977*
	Acidaminococcus	14.74	*0.296*	18.26	*0.157*	0.40	*0.963*	1.96	*0.910*
	Acidaminococcaceae_uncl.	1.25	*0.994*	2.01	*0.904*	0.98	*1.000*	1.06	*0.999*
	Succiniclasticum	1.53	*0.688*	1.60	*0.622*	1.70	*0.755*	0.97	*0.999*
	Phascolarctobacterium	1.04	*0.980*	1.21	*0.606*	0.82	*0.564*	0.98	*0.991*
**Clostridiales_uncl.**	-	0.99	*0.997*	0.98	*0.990*	1.09	*0.776*	1.19	*0.342*
**Clostridiaceae**	-	0.79	*0.970*	0.63	*0.914*	0.93	*0.999*	1.23	*0.991*
	Clostridium_sensu_stricto	0.79	*0.940*	0.63	*0.833*	0.93	*0.998*	1.23	*0.981*
**Enterococcaceae**	-	1.20	*1.000*	1.79	*0.995*	0.72	*1.000*	0.38	*0.999*
	Enterococcus	1.20	*0.999*	1.79	*0.990*	0.72	*1.000*	0.38	*0.998*
**Erysipelotrichaceae**	-	1.17	*0.965*	0.92	*0.992*	1.57	*0.666*	1.54	*0.688*
	Catenibacterium	1.20	*0.999*	1.39	*0.997*	1.56	*0.908*	0.23	*0.833*
	*Clostridium*_XVIII	2.72	*0.900*	1.09	*1.000*	2.80	*0.702*	3.19	*0.595*
	Coprobacillus	1.08	*1.000*	1.07	*1.000*	0.71	*1.000*	0.67	*1.000*
	Erysipelotrichaceae_uncl.	0.93	*0.992*	0.75	*0.918*	0.96	*0.999*	1.47	*0.925*
	Turicibacter	0.71	*1.000*	4.19	*0.993*	1.68	*0.995*	4.19	*0.895*
**Firmicutes_uncl.**	-	0.96	*0.932*	1.07	*0.757*	0.80	***0.085***	0.71	***0.006***
**Lachnospiraceae**	-	1.08	***0.000***	1.02	*0.561*	1.00	*1.000*	1.04	***0.057***
	Anaerostipes	0.86	*0.840*	1.02	*0.998*	2.31	***0.006***	2.25	***0.010***
	Blautia	1.52	***0.000***	1.21	***0.003***	0.95	*0.484*	0.97	*0.704*

	*Clostridium*_XlVa_uncl.	1.08	*0.987*	1.00	*1.000*	1.03	*0.992*	1.05	*0.976*
	*Clostridium*_XlVb_uncl.	1.15	*0.998*	0.76	*0.995*	0.96	*1.000*	0.61	*0.983*
	Coprococcus	0.97	*0.970*	0.96	*0.942*	0.80	*0.168*	0.70	***0.019***
	Dorea	0.96	*0.942*	0.83	*0.338*	1.13	*0.730*	0.79	*0.476*
	Lachnospira	0.96	*0.998*	1.83	*0.569*	0.45	*0.711*	1.13	*0.981*
	Lachnospiraceae_uncl.	0.88	***0.038***	0.92	*0.271*	1.01	*0.963*	1.13	***0.039***
	Howardella	0.98	*1.000*	1.68	*0.984*	0.40	*0.998*	0.52	*0.999*
	Roseburia	1.11	*0.134*	0.94	*0.504*	1.00	*1.000*	1.08	*0.414*
	Ruminococcus	0.95	*0.979*	1.33	*0.354*	0.90	*0.871*	1.26	*0.438*
**Lactobacillaceae**	-	1.30	*0.996*	0.17	*0.966*	0.77	*1.000*	1.42	*0.999*
	Lactobacillus	1.30	*0.991*	0.17	*0.932*	0.77	*0.999*	1.42	*0.997*
**Peptostreptococcaceae**	-	1.04	*0.994*	1.21	*0.874*	0.91	*0.963*	0.77	*0.777*
	*Clostridium*_XI	1.04	*0.988*	1.21	*0.763*	0.91	*0.925*	0.77	*0.597*
**Ruminococcaceae**	-	0.90	***0.000***	0.91	***0.000***	0.99	*0.954*	0.91	***0.000***
	Butyricicoccus	1.04	*0.997*	0.89	*0.976*	1.10	*0.994*	0.90	*0.994*
	Faecalibacterium	0.67	***0.000***	0.75	***0.001***	0.91	*0.224*	0.82	***0.004***
	Flavonifractor	1.41	*0.950*	1.28	*0.978*	1.07	*0.999*	0.90	*0.996*
	Gemmiger	0.96	*0.977*	0.75	*0.458*	0.89	*0.885*	1.09	*0.916*
	*Clostridium*_IV_uncl.	0.49	*0.861*	0.89	*0.993*	0.68	*0.983*	0.45	*0.953*
	Oscillibacter	0.61	*0.878*	1.28	*0.935*	2.12	*0.467*	2.16	*0.443*
	Ruminococcaceae_uncl.	0.96	*0.263*	0.96	*0.241*	0.97	*0.616*	0.91	***0.009***
	Ruminococcus	1.08	*0.758*	1.02	*0.977*	1.10	*0.725*	0.90	*0.763*
**Streptococcaceae**	-	0.78	*0.958*	0.94	*0.997*	0.74	*0.923*	0.91	*0.990*
	Lactococcus	0.82	*0.998*	1.15	*0.999*	0.59	*0.999*	1.29	*0.999*
	Streptococcus	0.77	*0.937*	0.89	*0.986*	0.75	*0.869*	0.89	*0.972*
**Veillonellaceae**	-	1.76	***0.026***	1.41	*0.303*	0.89	*0.860*	1.24	*0.537*
	Dialister	1.92	***0.001***	1.42	*0.172*	0.93	*0.926*	1.33	*0.211*
	Megamonas	2.42	*0.998*	3.15	*0.995*	2.40	*0.999*	0.81	*1.000*
	Megasphaera	0.85	*0.997*	1.22	*0.993*	0.59	*0.918*	0.76	*0.970*
	Mitsuokella	0.65	*0.999*	0.80	*1.000*	1.13	*1.000*	1.78	*0.998*
	Veillonella	0.82	*0.999*	1.68	*0.992*	0.53	*0.999*	0.78	*1.000*
**Phylum Proteobacteria**									
**Alphaproteobacteria_uncl.**	-	1.00	*1.000*	1.00	*1.000*	0.63	*0.991*	1.41	*0.989*
**Desulfovibrionaceae**	-	1.30	*0.987*	1.66	*0.939*	0.73	*0.990*	1.02	*1.000*
	Desulfovibrio	1.32	*0.980*	1.67	*0.916*	0.69	*0.999*	1.13	*1.000*
	Desulfovibrionaceae_uncl.	1.24	*0.999*	1.61	*0.996*	0.74	*0.988*	0.99	*1.000*
**Enterobacteriaceae**	-	0.69	*0.953*	1.17	*0.985*	0.58	*0.676*	0.90	*0.976*
	Escherichia/Shigella	0.67	*0.925*	1.27	*0.950*	0.59	*0.715*	1.01	*1.000*
	Klebsiella	0.81	*0.999*	0.72	*0.997*	0.56	*0.895*	0.70	*0.947*
**Sutterellaceae**	-	0.84	*1.000*	1.05	*1.000*	3.41	*0.665*	0.88	*0.999*
	Parasutterella	1.09	*1.000*	0.65	*1.000*	1.04	*1.000*	0.59	*0.997*
	Sutterella	0.77	*0.999*	1.12	*1.000*	4.60	*0.440*	1.03	*1.000*
**Oxalobacteraceae**	-	1.00	*1.000*	1.00	*1.000*	1.28	*0.999*	1.32	*0.999*
	Oxalobacteraceae_uncl.	1.00	*1.000*	1.00	*1.000*	1.28	*0.998*	1.32	*0.998*
**Pasteurellaceae**	-	0.70	*1.000*	0.22	*1.000*	1.79	*0.998*	1.22	*1.000*
	Haemophilus	0.70	*1.000*	0.22	*1.000*	1.79	*0.996*	1.22	*1.000*
**Phylum Spirochaetae**									
**Brachyspiraceae**	-	1.02	*1.000*	1.08	*1.000*	0.95	*1.000*	0.76	*0.998*
	Brachyspira	1.02	*1.000*	1.08	*0.999*	0.95	*1.000*	0.76	*0.996*
**Spirochaetaceae**	-	1.98	*1.000*	1.67	*1.000*	0.99	*1.000*	0.36	*0.999*
	Treponema	1.98	*0.999*	1.67	*1.000*	0.99	*1.000*	0.36	*0.999*
**Phylum Verrucomicrobia**									
**Verrucomicrobiaceae**	*Akkermansia*	1.11	*0.855*	1.07	*0.931*	1.35	*0.425*	1.20	*0.737*

Legend: A two-way repeated measures ANOVA with Dunnett's multiple comparison's test against V1 was performed to test for significant changes over time within each group (*p*-values ≤ 0.05 and < 0.1 are depicted in bold text). FC, fold-change; V, visit.
