# Supplementary material for: A yeast fermentate improves gastrointestinal discomfort and constipation by modulation of the gut microbiome: results from a randomized double-blind placebo-controlled pilot trial
Source: BMC Complement Altern Med. 2017 Sep 4;17:441. doi: 10.1186/s12906-017-1948-0 (PMC5584023; doi:10.1186/s12906-017-1948-0)
Supplement: Supplementary file 7 — Microbial taxa fold-changes (V2/V1 and V3/V1) in the severe subgroup. (PDF 554 kb) [file 12906_2017_1948_MOESM7_ESM.pdf]

Additional file 8 Microbial taxa fold-changes (V2/V1 and V3/V1) in the severe subgroup.

Taxa		Severe subgroup (n=54)							
		Placebo (n=27)				EpiCor (n=27)			
		V2/V1		V3/V1		V2/V1		V3/V1	
		FC	*p*-val.	FC	*p*-val.	FC	*p*-val.	FC	*p*-val.
**Phylum Actinobacteria**									
**Bifidobacteriaceae**	-	0.72	0.162	1.05	0.927	1.04	0.956	1.07	0.879
	Bifidobacterium	0.72	**0.032**	1.05	0.859	1.04	0.911	1.07	0.766
**Coriobacteriaceae**	-	1.06	0.928	1.07	0.904	1.21	0.637	0.96	0.981
	Asaccharobacter	1.41	0.982	1.42	0.982	1.40	0.967	1.34	0.976
	Collinsella	1.29	0.383	1.30	0.351	0.82	0.847	0.66	0.560
	Coriobacteriaceae_uncl.	0.61	0.620	0.73	0.789	1.84	0.383	1.43	0.769
	Eggerthella	2.60	1.000	1.87	1.000	1.00	1.000	0.00	1.000
	Enterorhabdus	1.33	0.993	0.64	0.991	0.51	0.953	0.63	0.973
	Slackia	0.95	1.000	0.38	0.964	3.20	0.854	0.80	0.999
**Propionibacteriaceae**	-	0.07	0.991	0.01	1.000	2.11	0.997	1.56	0.999
	Propionibacterium	0.07	0.983	0.01	0.999	2.11	0.993	1.56	0.998
**Phylum Bacteroidetes**									
**Bacteroidaceae**	-	0.84	**0.054**	0.89	0.216	1.15	0.118	1.14	0.161
	Bacteroides	0.84	**0.004**	0.89	**0.054**	1.15	**0.015**	1.14	**0.027**
**Bacteroidetes_uncl.**	-	1.31	0.949	0.86	0.989	0.96	0.999	0.89	0.989
**Bacteroidales_uncl.**	-	1.21	0.992	0.67	0.981	1.20	0.997	1.93	0.943
**Porphyromonadaceae**	-	0.86	0.549	1.03	0.979	1.13	0.513	0.96	0.926
	Barnesiella	0.83	0.883	0.84	0.892	1.39	0.227	1.31	0.371
	Butyricimonas	1.82	0.979	1.63	0.988	0.80	0.987	0.77	0.984
	Odoribacter	0.65	0.969	1.27	0.982	1.59	0.980	1.68	0.973
	Porphyromonadaceae_uncl.	0.93	0.973	1.17	0.836	1.07	0.944	0.85	0.779
	Parabacteroides	0.93	0.969	0.98	0.998	0.84	0.893	0.54	0.415
**Prevotellaceae**	-	0.77	0.539	1.03	0.987	1.43	0.138	1.01	0.999
	Alloprevotella	1.33	0.987	2.11	0.860	0.71	0.991	0.79	0.996
	Hallella	1.00	1.000	1.00	1.000	0.00	0.924	0.00	0.924
	Paraprevotella	0.02	0.624	0.01	0.619	1.71	0.681	0.14	0.575
	Prevotella	0.80	0.603	1.11	0.856	1.68	**0.039**	1.35	0.389
	Prevotellaceae_uncl.	1.41	0.971	1.21	0.992	0.94	0.998	1.19	0.982
**Rikenellaceae**	-	1.15	0.524	0.74	0.154	0.55	**0.035**	0.64	0.113
	Alistipes	1.15	0.288	0.74	**0.029**	0.55	**0.001**	0.64	**0.013**
**Phylum Firmicutes**									
**Acidaminococcaceae**	-	1.51	**0.099**	1.70	**0.017**	1.06	0.975	1.07	0.965
	Acidaminococcus	14.95	0.174	18.50	**0.070**	0.00	1.000	0.00	1.000
	Acidaminococcaceae_uncl.	1.15	0.999	1.85	0.953	1.53	0.977	1.45	0.984
	Succiniclasticum	1.62	0.614	1.46	0.760	3.09	0.646	0.90	0.999
	Phascolarctobacterium	1.06	0.972	1.21	0.669	0.83	0.712	1.05	0.969
**Clostridiales_uncl.**	-	0.82	0.564	1.04	0.969	1.16	0.594	1.19	0.488
**Clostridiaceae**	-	0.75	0.956	0.66	0.918	0.92	0.999	0.84	0.996
	Clostridium_sensu_stricto	0.75	0.914	0.66	0.843	0.92	0.998	0.84	0.992
**Enterococcaceae**	-	1.02	1.000	1.28	0.999	0.81	1.000	0.59	1.000
	Enterococcus	1.02	1.000	1.28	0.999	0.81	1.000	0.59	1.000
**Erysipelotrichaceae**	-	1.41	0.910	1.12	0.991	1.61	0.895	2.13	0.686
	Catenibacterium	1.16	0.999	1.17	0.999	0.27	0.999	0.27	0.999
	*Clostridium*_XVIII	4.08	0.932	1.79	0.995	3.16	0.660	2.95	0.712
	Coprobacillus	1.45	1.000	1.66	0.999	0.40	0.999	0.74	1.000
	Erysipelotrichaceae_uncl.	1.17	0.985	1.01	1.000	0.75	0.993	0.96	1.000
	Turicibacter	1.00	1.000	1.29	1.000	0.70	1.000	8.85	0.870
**Firmicutes_uncl.**	-	1.04	0.936	1.15	0.386	0.82	0.227	0.59	**0.002**
**Lachnospiraceae**	-	1.09	**0.000**	0.98	0.593	1.03	0.294	1.09	**0.001**
	Anaerostipes	0.89	0.901	0.63	0.318	2.81	**0.001**	2.69	**0.003**
	Blautia	1.86	**0.000**	1.36	**0.000**	1.00	0.999	1.05	0.585

	*Clostridium*_XlVa_uncl.	0.92	0.991	1.08	0.991	1.10	0.959	0.99	1.000
	*Clostridium*_XlVb_uncl.	0.98	1.000	0.83	0.998	1.06	1.000	0.62	0.988
	Coprococcus	0.86	0.505	0.78	0.195	0.78	0.257	0.71	0.104
	Dorea	1.03	0.979	0.95	0.952	1.08	0.935	0.75	0.510
	Lachnospira	0.62	0.968	2.29	0.691	0.29	0.653	1.10	0.990
	Lachnospiraceae_uncl.	0.91	0.296	0.89	0.189	0.86	0.152	1.07	0.581
	Howardella	1.04	1.000	1.98	0.986	0.40	0.998	0.52	0.999
	Roseburia	0.92	0.432	0.75	**0.002**	1.15	0.197	1.21	**0.043**
	Ruminococcus	1.13	0.942	1.97	**0.070**	1.21	0.802	1.41	0.439
**Lactobacillaceae**	-	1.29	0.994	0.17	0.950	0.84	1.000	1.04	1.000
	Lactobacillus	1.29	0.987	0.17	0.902	0.84	1.000	1.04	1.000
**Peptostreptococcaceae**	-	1.01	1.000	1.22	0.855	0.60	0.679	0.79	0.894
	*Clostridium*_XI	1.01	1.000	1.22	0.733	0.60	0.456	0.79	0.793
**Ruminococcaceae**	-	0.90	**0.000**	0.92	**0.004**	0.93	**0.031**	0.89	**0.000**
	Butyricicoccus	0.66	0.819	0.67	0.820	1.12	0.994	0.78	0.980
	Faecalibacterium	0.64	**0.000**	0.70	**0.001**	0.87	**0.086**	0.77	**0.001**
	Flavonifractor	1.74	0.930	1.49	0.969	1.38	0.984	1.28	0.991
	Gemmiger	0.82	0.727	0.67	0.358	0.72	0.640	0.95	0.985
	*Clostridium*_IV_uncl.	0.40	0.809	0.89	0.993	0.35	0.958	0.36	0.959
	Oscillibacter	0.53	0.834	0.86	0.984	1.87	0.844	2.53	0.606
	Ruminococcaceae_uncl.	0.98	0.760	1.04	0.445	0.92	0.177	0.92	0.182
	Ruminococcus	1.13	0.618	0.95	0.935	1.15	0.626	0.89	0.782
**Streptococcaceae**	-	0.98	1.000	1.10	0.996	0.70	0.943	0.98	1.000
	Lactococcus	0.78	0.996	1.10	0.999	0.44	0.999	1.24	1.000
	Streptococcus	1.07	0.998	1.10	0.997	0.71	0.902	0.96	0.998
**Veillonellaceae**	-	1.82	**0.014**	1.48	0.202	0.95	0.980	1.34	0.371
	Dialister	1.98	**0.000**	1.50	**0.085**	1.00	1.000	1.43	0.103
	Megamonas	3.26	0.996	2.12	0.999	2.40	0.999	0.81	1.000
	Megasphaera	0.74	0.990	1.19	0.995	0.53	0.942	0.77	0.986
	Mitsuokella	0.40	0.999	1.03	1.000	1.49	1.000	1.34	1.000
	Veillonella	0.87	1.000	2.05	0.990	0.40	1.000	0.62	1.000
**Phylum Proteobacteria**									
**Alphaproteobacteria_uncl.**	-	1.00	1.000	1.00	1.000	0.71	0.997	1.93	0.967
**Desulfovibrionaceae**	-	1.88	0.993	4.18	0.915	0.80	0.996	1.01	1.000
	Desulfovibrio	2.88	0.989	7.83	0.860	1.28	1.000	2.02	0.999
	Desulfovibrionaceae_uncl.	1.12	1.000	1.41	0.999	0.77	0.990	0.94	0.999
**Enterobacteriaceae**	-	0.50	0.911	1.23	0.981	0.66	0.838	0.77	0.926
	Escherichia/Shigella	0.46	0.871	1.33	0.947	0.63	0.772	0.83	0.945
	Klebsiella	0.64	0.997	0.77	0.999	0.75	0.991	0.59	0.974
**Sutterellaceae**	-	0.94	1.000	1.44	0.999	1.26	0.998	0.95	1.000
	Parasutterella	1.07	1.000	0.65	1.000	1.10	1.000	0.62	0.998
	Sutterella	0.86	1.000	1.72	0.997	1.44	0.997	1.27	0.999
**Oxalobacteraceae**	-	1.00	1.000	1.00	1.000	0.00	0.997	0.00	0.997
	Oxalobacteraceae_uncl.	1.00	1.000	1.00	1.000	0.00	0.994	0.00	0.994
**Pasteurellaceae**	-	0.70	1.000	0.19	1.000	1.76	0.998	1.20	1.000
	Haemophilus	0.70	1.000	0.19	1.000	1.76	0.996	1.20	1.000
**Phylum Spirochaetae**									
**Brachyspiraceae**	-	1.00	1.000	1.56	0.997	1.01	1.000	0.76	0.999
	Brachyspira	1.00	1.000	1.56	0.994	1.01	1.000	0.76	0.999
**Spirochaetaceae**	-	1.83	1.000	1.48	1.000	1.78	1.000	0.78	1.000
	Treponema	1.83	0.999	1.48	1.000	1.78	1.000	0.78	1.000
**Phylum Verrucomicrobia**									
**Verrucomicrobiaceae**	*Akkermansia*	1.22	0.707	1.23	0.680	0.77	0.825	0.84	0.908

Legend: A two-way repeated measures ANOVA with Dunnett's multiple comparison's test against V1 was performed to test for significant changes over time within each group (p-values ≤ 0.05 and < 0.1 are depicted in bold text). FC, fold-change; V, visit.
