# Supplementary material for: A yeast fermentate improves gastrointestinal discomfort and constipation by modulation of the gut microbiome: results from a randomized double-blind placebo-controlled pilot trial
Source: BMC Complement Altern Med. 2017 Sep 4;17:441. doi: 10.1186/s12906-017-1948-0 (PMC5584023; doi:10.1186/s12906-017-1948-0)
Supplement: Supplementary file 8 — Microbial taxa fold-changes (V2/V1 and V3/V1) in the moderate subgroup. (PDF 551 kb) [file 12906_2017_1948_MOESM8_ESM.pdf]

Additional file 9 Microbial taxa fold-changes (V2/V1 and V3/V1) in the moderate subgroup.

Taxa		Moderate subgroup (n=25)							
		Placebo (n=13)				EpiCor (n=12)			
		V2/V1		V3/V1		V2/V1		V3/V1	
		FC	*p*-val.	FC	*p*-val.	FC	*p*-val.	FC	*p*-val.
**Phylum Actinobacteria**									
**Bifidobacteriaceae**	-	1.13	0.672	0.84	0.561	0.71	0.213	0.90	0.825
	Bifidobacterium	1.13	0.443	0.84	0.310	0.71	**0.053**	0.90	0.683
**Coriobacteriaceae**	-	1.60	0.743	2.11	0.377	0.82	0.583	1.26	0.326
	Asaccharobacter	0.45	1.000	17.36	0.748	1.42	0.937	2.22	0.585
	Collinsella	1.90	0.752	1.67	0.855	0.74	0.734	0.61	0.503
	Coriobacteriaceae_uncl.	1.30	0.986	2.71	0.642	0.99	1.000	2.95	**0.001**
	Eggerthella	1.00	1.000	1.00	1.000	0.53	0.983	0.52	0.983
	Enterorhabdus	1.36	0.992	0.35	0.973	0.43	0.994	0.66	0.998
	Slackia	2.08	0.995	1.69	0.998	0.62	0.799	0.17	0.370
**Propionibacteriaceae**	-	1.00	1.000	1.00	1.000	9.67	0.994	0.33	1.000
	Propionibacterium	1.00	1.000	1.00	1.000	9.67	0.988	0.33	1.000
**Phylum Bacteroidetes**									
**Bacteroidaceae**	-	1.50	**0.011**	1.73	**0.000**	0.88	0.218	0.97	0.886
	Bacteroides	1.50	**0.000**	1.73	**0.000**	0.88	**0.055**	0.97	0.785
**Bacteroidetes_uncl.**	-	1.25	0.991	1.69	0.936	1.04	0.997	0.56	0.694
**Bacteroidales_uncl.**	-	2.51	0.966	1.80	0.990	1.19	1.000	2.01	0.991
**Porphyromonadaceae**	-	0.54	**0.000**	0.53	**0.000**	0.93	0.928	1.13	0.790
	Barnesiella	0.48	**0.051**	0.51	**0.065**	1.45	0.840	1.71	0.652
	Butyricimonas	0.93	1.000	1.34	0.999	0.58	0.915	1.07	0.998
	Odoribacter	0.96	1.000	0.78	0.998	0.80	0.997	0.51	0.979
	Porphyromonadaceae_uncl.	0.85	0.803	0.74	0.499	1.13	0.951	1.06	0.988
	Parabacteroides	0.29	**0.004**	0.34	**0.008**	0.60	0.654	1.02	0.999
**Prevotellaceae**	-	0.86	0.834	0.88	0.884	1.01	0.999	1.43	0.326
	Alloprevotella	0.73	0.989	0.55	0.970	1.12	1.000	1.06	1.000
	Hallella	1.00	1.000	1.00	1.000	1.00	1.000	1.00	1.000
	Paraprevotella	0.40	1.000	0.20	1.000	0.50	1.000	1.50	1.000
	Prevotella	0.86	0.802	0.94	0.961	0.96	0.990	1.63	0.128
	Prevotellaceae_uncl.	0.93	0.998	0.79	0.981	1.13	0.977	1.03	0.999
**Rikenellaceae**	-	0.91	0.972	1.37	0.598	1.11	0.869	1.22	0.554
	Alistipes	0.91	0.942	1.37	0.351	1.11	0.756	1.22	0.318
**Phylum Firmicutes**									
**Acidaminococcaceae**	-	1.06	0.989	1.46	0.600	0.72	0.336	0.99	0.998
	Acidaminococcus	0.00	1.000	0.00	1.000	0.40	0.812	1.96	0.598
	Acidaminococcaceae_uncl.	1.45	0.993	2.34	0.941	0.40	0.886	0.66	0.960
	Succiniclasticum	1.16	0.996	2.18	0.801	0.84	0.969	1.01	1.000
	Phascolarctobacterium	1.00	1.000	1.20	0.889	0.81	0.729	0.80	0.701
**Clostridiales_uncl.**	-	1.26	0.393	0.89	0.837	0.89	0.861	1.20	0.617
**Clostridiaceae**	-	0.99	1.000	0.45	0.988	0.96	1.000	3.76	0.870
	Clostridium_sensu_stricto	0.99	1.000	0.45	0.975	0.96	1.000	3.76	0.758
**Enterococcaceae**	-	2.11	0.999	4.38	0.993	0.64	0.999	0.17	0.997
	Enterococcus	2.11	0.998	4.38	0.984	0.64	0.999	0.17	0.994
**Erysipelotrichaceae**	-	0.88	0.988	0.68	0.921	1.54	0.513	1.09	0.981
	Catenibacterium	4.25	1.000	16.00	0.996	1.68	0.525	0.22	0.438
	*Clostridium*_XVIII	1.91	0.964	0.67	0.995	1.15	1.000	4.30	0.799
	Coprobacillus	0.72	1.000	0.48	0.999	1.48	1.000	0.48	1.000
	Erysipelotrichaceae_uncl.	0.66	0.892	0.47	0.760	1.19	0.984	2.02	0.627
	Turicibacter	0.66	1.000	4.68	0.977	2.37	0.961	0.89	1.000
**Firmicutes_uncl.**	-	0.78	0.506	0.86	0.755	0.76	0.239	1.04	0.954
**Lachnospiraceae**	-	1.06	**0.096**	1.09	**0.009**	0.93	**0.006**	0.94	**0.011**
	Anaerostipes	0.72	0.928	2.78	**0.080**	0.67	0.886	0.83	0.969
	Blautia	1.01	0.981	0.98	0.959	0.84	**0.023**	0.77	**0.001**

	*Clostridium*_XlVa_uncl.	1.40	0.889	0.84	0.981	0.92	0.948	1.14	0.853
	*Clostridium*_XlVb_uncl.	1.60	0.993	0.58	0.997	0.60	0.995	0.56	0.994
	Coprococcus	1.36	0.442	1.60	0.124	0.86	0.591	0.67	**0.076**
	Dorea	0.85	0.668	0.64	0.106	1.29	0.549	0.89	0.917
	Lachnospira	1.20	0.964	1.49	0.808	1.05	0.999	1.22	0.985
	Lachnospiraceae_uncl.	0.82	**0.060**	0.99	0.983	1.26	**0.000**	1.22	**0.001**
	Howardella	0.91	1.000	1.34	0.997	1.00	1.000	1.00	1.000
	Roseburia	1.56	**0.000**	1.36	**0.004**	0.70	**0.002**	0.80	**0.053**
	Ruminococcus	0.80	0.688	0.78	0.624	0.47	**0.047**	1.06	0.956
**Lactobacillaceae**	-	0.00	1.000	0.00	1.000	0.75	0.998	1.54	0.993
	Lactobacillus	0.00	1.000	0.00	1.000	0.75	0.997	1.54	0.986
**Peptostreptococcaceae**	-	1.20	0.987	1.13	0.994	1.50	0.452	0.73	0.789
	*Clostridium*_XI	1.20	0.972	1.13	0.988	1.50	0.217	0.73	0.625
**Ruminococcaceae**	-	0.92	**0.048**	0.89	**0.007**	1.15	**0.000**	0.95	0.256
	Butyricicoccus	2.42	0.578	1.71	0.869	1.02	1.000	1.31	0.983
	Faecalibacterium	0.72	**0.026**	0.83	0.225	1.09	0.724	1.00	1.000
	Flavonifractor	1.02	1.000	1.02	1.000	0.73	0.968	0.49	0.889
	Gemmiger	1.33	0.731	0.96	0.994	1.28	0.676	1.43	0.414
	*Clostridium*_IV_uncl.	0.95	1.000	0.90	0.999	1.54	0.981	0.70	0.994
	Oscillibacter	0.95	1.000	3.03	0.667	2.41	0.246	1.74	0.656
	Ruminococcaceae_uncl.	0.92	0.181	0.80	**0.000**	1.06	0.204	0.88	**0.002**
	Ruminococcus	0.98	0.992	1.17	0.663	0.96	0.981	0.94	0.971
**Streptococcaceae**	-	0.57	0.887	0.77	0.964	0.82	0.976	0.78	0.963
	Lactococcus	1.53	1.000	2.11	1.000	0.77	1.000	1.35	0.999
	Streptococcus	0.56	0.770	0.74	0.914	0.83	0.960	0.74	0.910
**Veillonellaceae**	-	1.46	0.855	1.10	0.993	0.59	0.678	0.80	0.906
	Dialister	1.60	0.724	1.03	0.999	0.55	0.661	0.75	0.879
	Megamonas	0.19	1.000	5.94	0.994	1.00	1.000	1.00	1.000
	Megasphaera	1.32	0.998	1.31	0.998	0.71	0.971	0.73	0.975
	Mitsuokella	1.02	1.000	0.46	0.999	0.43	0.999	2.63	0.994
	Veillonella	0.76	0.999	1.13	1.000	0.63	0.998	0.90	1.000
**Phylum Proteobacteria**									
**Alphaproteobacteria_uncl.**	-	1.00	1.000	1.00	1.000	0.45	0.990	0.20	0.978
**Desulfovibrionaceae**	-	1.16	0.993	1.02	1.000	0.45	0.991	1.06	1.000
	Desulfovibrio	1.13	0.991	0.94	0.998	0.48	0.991	0.81	0.999
	Desulfovibrionaceae_uncl.	1.46	0.999	1.97	0.996	0.38	0.998	1.65	0.998
**Enterobacteriaceae**	-	1.13	0.997	1.05	1.000	0.37	0.694	1.24	0.948
	Escherichia/Shigella	1.10	0.998	1.13	0.996	0.31	0.920	2.28	0.758
	Klebsiella	1.30	0.999	0.57	0.999	0.40	0.716	0.79	0.959
**Sutterellaceae**	-	0.68	1.000	0.41	0.999	6.46	0.149	0.79	0.997
	Parasutterella	0.00	1.000	0.00	1.000	0.56	1.000	0.36	0.999
	Sutterella	0.67	0.999	0.41	0.997	7.06	**0.025**	0.84	0.997
**Oxalobacteraceae**	-	1.00	1.000	1.00	1.000	2.51	0.965	2.58	0.961
	Oxalobacteraceae_uncl.	1.00	1.000	1.00	1.000	2.51	0.930	2.58	0.924
**Pasteurellaceae**	-	0.00	1.000	0.00	1.000	0.00	1.000	0.00	1.000
	Haemophilus	0.00	1.000	0.00	1.000	0.00	1.000	0.00	1.000
**Phylum Spirochaetae**									
**Brachyspiraceae**	-	1.04	1.000	0.75	0.997	0.86	0.999	0.76	0.998
	Brachyspira	1.04	1.000	0.75	0.995	0.86	0.999	0.76	0.996
**Spirochaetaceae**	-	2.50	1.000	2.33	1.000	0.66	1.000	0.18	0.997
	Treponema	2.50	1.000	2.33	1.000	0.66	0.999	0.18	0.994
**Phylum Verrucomicrobia**									
**Verrucomicrobiaceae**	*Akkermansia*	0.93	0.973	0.82	0.821	2.42	**0.000**	1.88	**0.036**

Legend: A two-way repeated measures ANOVA with Dunnett's multiple comparison's test against V1 was performed to test for significant changes over time within each group (p-values ≤ 0.05 and < 0.1 are depicted in bold text). FC, fold-change; V, visit.
